# Multi-Stage Bioprocessing of Bee Pollen by LAB Fermentation and Enzymatic Hydrolysis: Enhancing Phenolic and Flavonoid Content and Bioactivity

**DOI:** 10.3390/foods15173056

**Published:** 2026-08-28

**Authors:** Vilma Kaškonienė, Vaida Damulienė, Paulius Kaškonas

**Affiliations:** 1 Instrumental Analysis Open Access Centre, Vytautas Magnus University, Akademija, LT-53361 Kaunas, Lithuania; 2 Institute of Metrology, Electrical and Electronics Engineering Faculty, Kaunas University of Technology, LT-51368 Kaunas, Lithuania

**Keywords:** multi-stage bioprocessing, lactic acid fermentation, enzymatic hydrolysis, antioxidants, bioactivity

## Abstract

Bee pollen is a rich natural source of phenolic compounds and other bioactive constituents; however, the resistant pollen cell wall restricts the release of these compounds. This study investigated the effect of multi-stage bioprocessing involving bacterial fermentation, spontaneous fermentation, and enzymatic hydrolysis on the recovery of bioactive compounds from ten bee pollen collected from different regions of Lithuania. First-stage treatments included fermentation with *Lactobacillus acidophilus*, *L. rhamnosus*, *L. reuteri*, *Bifidobacterium animalis*, spontaneous fermentation, or enzymatic hydrolysis using Viscozyme^®^ L, cellulase, or Clara-diastase. Second-stage bioprocessing combined the initial treatment with either bacterial fermentation or enzymatic hydrolysis. Total phenolic content (TPC), total flavonoid content (TFC), radical scavenging activity (RSA), and inhibition of heat-induced bovine serum albumin (BSA) denaturation (BSA-DI) were determined using spectrophotometric assays. Statistical analysis demonstrated that multi-stage bioprocessing significantly enhanced the chemical composition and bioactivity of bee pollen compared with single-stage treatments. The greatest improvements were achieved when enzymatic hydrolysis preceded bacterial fermentation, resulting in increases of up to 5.0-, 4.2-, 6.3-, and 5.3-fold in TPC, TFC, RSA, and BSA-DI activity, respectively. These findings suggest that combining enzymatic hydrolysis with bacterial fermentation may be a promising approach for improving the recovery of bioactive compounds and selected functional properties of bee pollen.

## 1. Introduction

Bee pollen plays a crucial role in plant reproduction and is a natural substance characterized by intricate chemical, structural, and microbiological features. Each pollen grain is composed of four principal components—exine, intine, pollenkitt, and cytoplasm—that collectively safeguard the genetic material during dispersal and enable successful fertilization [1,2,3]. The exine, built from the highly durable biopolymer sporopollenin, ensures structural stability and protects the grain against mechanical damage, desiccation, and ultraviolet light [4]. Directly beneath it lies the intine, a more pliable layer composed mainly of cellulose and pectin, which is essential for pollen germination and the elongation of the pollen tube [5,6]. The outer surface of the exine is covered by a lipid-rich material known as pollenkitt, which enhances attachment to pollinators and protects the grain from environmental challenges [7,8]. The cytoplasm contains the genetic material, along with organelles and enzymes required for pollen tube formation and the fertilization process [1,9].

From a chemical standpoint, bee pollen constitutes a complex matrix rich in proteins, lipids, carbohydrates, vitamins, minerals, and phenolic compounds. The protein fraction, which ranges from approximately 4% to 60%, supplies essential amino acids indispensable for human nutrition and metabolic homeostasis [10,11]. The lipid component—comprising essential fatty acids and phytosterols—is associated with antibacterial and antifungal activities, as well as cardioprotective properties [12,13]. Carbohydrates function primarily as an immediate energy source and provide dietary fiber, contributing to the maintenance of gastrointestinal health [10]. Furthermore, bee pollen contains a broad spectrum of vitamins and minerals, including B-complex vitamins, vitamin C, potassium, calcium, and magnesium, which are integral to numerous physiological pathways [6]. Phenolic compounds, notably flavonoids such as quercetin and kaempferol, exhibit pronounced antioxidant activity and influence the characteristic color and aroma of the pollen [14,15,16].

Bee pollen also hosts a diverse microbiota, including lactic acid bacteria, yeasts, and fungi, which influence its preservation and bioactivity [17,18]. Beneficial bacteria, such as *Lacticaseibacillus* and *Bifidobacterium*, produce antimicrobial compounds and support pollen stability [19,20], whereas certain fungi, including *Aspergillus* and *Penicillium*, may produce mycotoxins, highlighting the need for microbiological control [21,22].

The therapeutic potential of bee pollen is linked to its diverse bioactive compounds. Studies have reported anti-inflammatory, antimicrobial, antioxidant, anti-carcinogenic, anti-allergic, hepatoprotective, and cardioprotective effects [10,23,24]. These properties are primarily attributed to polyphenols, flavonoids, fatty acids, and phytosterols, which modulate immune responses, neutralize free radicals, and inhibit microbial growth. Despite extensive in vitro evidence, further research is required to confirm these effects in clinical settings.

In summary, bee pollen is a nutritionally rich and biologically active natural product with structural, chemical, and microbiological complexity. Its diverse composition underlies a wide range of potential health benefits, making it a promising candidate for functional foods, nutraceuticals, and therapeutic applications.

Pre-treatment of bee pollen significantly enhances its nutritional and bioactive profile by facilitating the release of intracellular constituents from the recalcitrant pollen wall. Fermentation with microorganisms, such as *Lactobacillus* or *Saccharomyces*, has been shown to improve antimicrobial efficacy, flavonoid concentrations, and antioxidant capacity [25,26,27]. Similarly, enzymatic hydrolysis utilizing cellulase, protease, or amylase increases antioxidant activity, protein and amino acid yields, carbohydrate levels, and overall digestibility [28,29,30].

Physical methods also offer distinct advantages: ultrasonication promotes the liberation of polyphenols, proteins, and fatty acids, although it may concurrently reduce sugar, mineral, and carotenoid content [31,32,33,34]. Microwave processing has been found to elevate fatty acid, protein, and vitamin C levels with minimal impact on polyphenolic profiles [35,36]. Furthermore, steam explosion increases nutrient density while effectively reducing microbial loads [37]. Mechanical grinding also further enhances the bioavailability of proteins, lipids, and phenolic compounds by liberating bound nutrients [38,39]. Collectively, these processing strategies may modify functional attributes of bee pollen, supporting further investigation of their potential applications in food and nutraceutical products.

The aim of this study was to evaluate the effects of single- and multi-stage bioprocessing involving LAB fermentation and enzymatic hydrolysis on the phenolic content, flavonoid content, radical scavenging activity, and inhibition of heat-induced bovine serum albumin denaturation of bee pollen collected in ten different regions in Lithuania. A partially related approach was reported by Xue et al. [40], who investigated tea bee pollen subjected to enzymatic digestion with a cellulase-pectinase complex followed by fermentation with *L. reuteri*, primarily focusing on the effects of the processed pollen on the growth and gut health of *Apis cerana cerana* workers. However, the sequential application of bacterial or spontaneous fermentation and enzymatic hydrolysis in both possible orders, using different bacterial strains and enzyme preparations, has not been systematically investigated for enhancing the recovery of bioactive compounds and related in vitro activities of bee pollen.

The hypothesis was that combining bacterial fermentation and enzymatic hydrolysis in a sequential manner may enhance the recovery of phenolic and flavonoid compounds and improve the measured antioxidant and BSA-denaturation inhibition activities compared with single-stage treatments.

## 2. Materials and Methods

### 2.1. Samples

**Figure 1:**
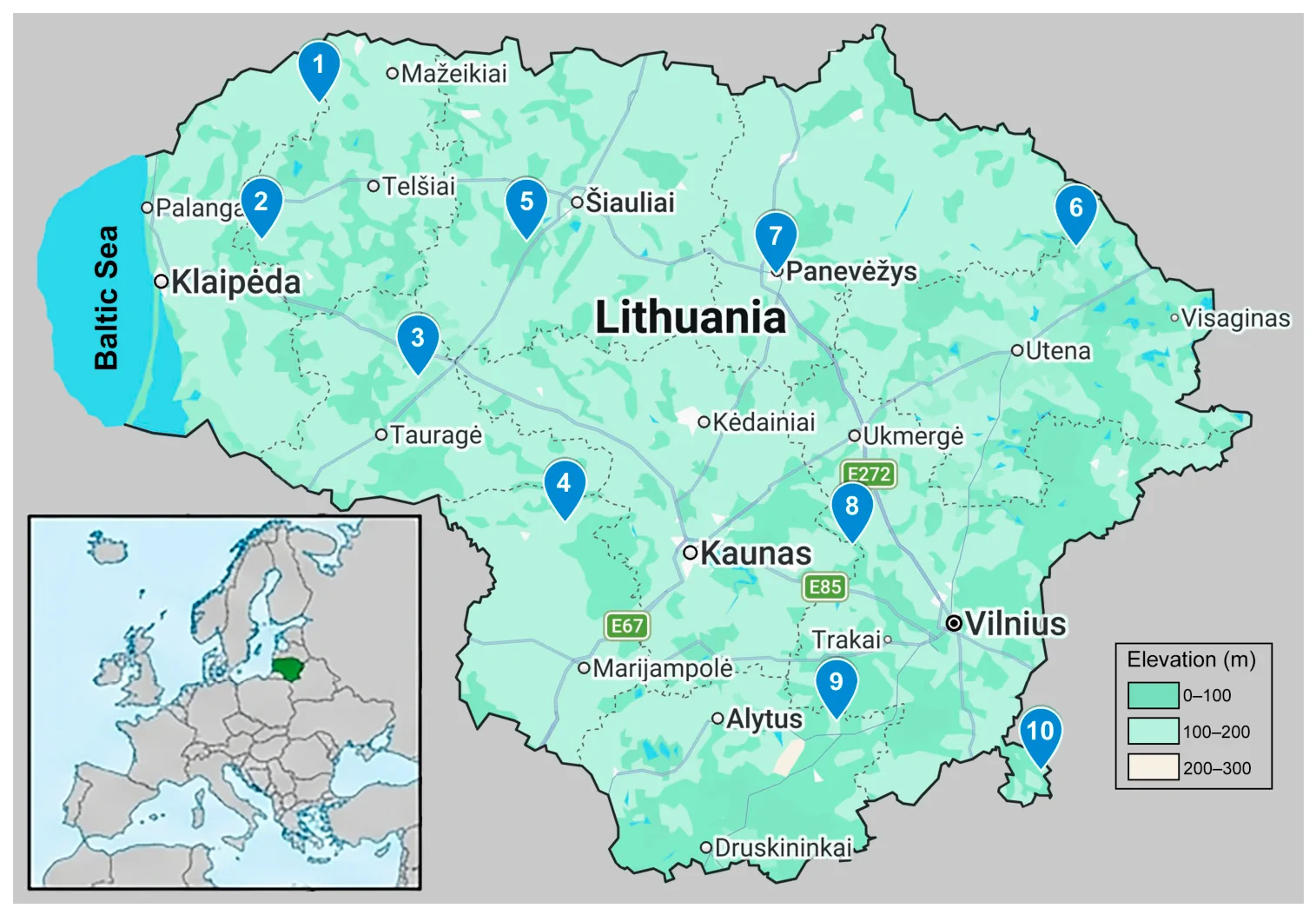
Geographical distribution of the bee pollen collection sites in Lithuania. Sampling sites are indicated on the map. Location numbers correspond to the sample numbers in Table 1.

Bee pollen samples collected from April to June were obtained from various Lithuanian counties. The exact locations are presented in Table 1 and shown on the map in Figure 1. Sampling sites were selected to ensure representative coverage of the geographical and climatic conditions across Lithuania. Dried samples were retained in a refrigerator at +5 °C for a maximum of six months according to recommendations described in ISO 24382:2023 [41]. Prior to analysis and extract preparation, the samples were homogenized using a pestle and porcelain mortar.

### 2.2. Reagents and Equipment

De Man, Rogosa, and Sharpe (MRS) broth and agar (Agar-Agar) (Biolife Italiana, Milan, Italy); phosphate-buffered saline (PBS) (VWR, Leuven, Belgium); methanol (≥99.8%) and acetonitrile (≥99.8%) (Merck, Darmstadt, Germany); Folin–Ciocalteu reagent (Merck Group, Darmstadt, Germany); aluminium chloride (≥98.0%) and hexamethylenetetramine (≥99.0%) (Carl Roth, Karlsruhe, Germany); sodium acetate trihydrate (99.9%) (ChemPur, Piekary Śląskie, Poland); sodium carbonate (>96%) and bovine serum albumin (Acros Organics, Geel, Belgium); rutin (95.0%), salicylic acid (≥99.0%) and DPPH (99.0%) (Sigma-Aldrich, Taufkirchen, Germany).

The following bacteria were used: *Lacticaseibacillus rhamnosus* GG (ATCC 53103) (Gefilus, Valio Ltd., Helsinki, Finland), *Lactobacillus acidophilus* (DSM 13241) (20 milliards, Gadot Biochemical Industries Ltd., Haifa, Israel), *Limosilactobacillus reuteri* (DSM 27938) (BioGaia Protectis, TwoPac AB, Eslöv, Sweden), and *Bifidobacterium animalis* (DSM 15954) (Biofarma spa, Mereto di Tomba, Italy).

Enzymes such as Viscozyme^®^ L (a mixture of cell wall degrading enzymes, including arabanase, cellulase, β-glucanase, hemicellulase and xylanase; from *Aspergillus* sp.; ≥100 FBG U/g (β-glucanase activity)); cellulase (from *Aspergillus niger*; ≥0.3 U/mg (cellulase activity)), and Clara-diastase (a mixture of α-amylase, cellulase, invertase, peptidase, phosphatase and sulfatase; ≥35 U/mg (maltose-releasing activity)) were from Sigma-Aldrich (Taufkirchen, Germany).

### 2.3. Solid-State Fermentation

For solid-state fermentation the following bacterial strains were used: *Lacticaseibacillus rhamnosus* GG (ATCC 53103), *Lactobacillus acidophilus* (DSM 13241), *Limosilactobacillus reuteri* (DSM 27938), *Bifidobacterium animalis* (DSM 15954). These bacterial strains were reactivated from the respective commercial probiotic preparations by spreading 100 µL of each bacterial suspension evenly onto the surface of solid MRS medium supplemented with agar in Petri dishes, using a sterile spreader and the surface-spreading method as described by Kaškonienė et al. [42]. The prepared bacterial samples were incubated in an incubator (Biosan, Riga, Latvia) at +37 °C for 48 h. After the incubation period, randomly selected colonies from the grown bacterial cultures were chosen for further experimental procedures. Thus, only isolated bacterial colonies were transferred to the fermentation medium, and the other ingredients present in the commercial probiotic formulations were not included.

Bacterial fermentation using *L. rhamnosus*, *B. animalis*, *L. acidophilus*, *L. reuteri*, and spontaneous fermentation (i.e., without the addition of bacteria) was conducted according to the method described by Kaškonienė et al. [42], with minor modifications. The samples were prepared in 4 mL vials by hydrating 1 g of homogenized bee pollen sample with 200 µL of sterile distilled water for 2 h. The lactic acid bacteria culture was grown to an optical density (measured at 600 nm) of 0.730 prior to incubation. This OD value was used as the criterion for standardizing the bacterial inoculum across the different fermentation treatments. Subsequently, a solution consisting of 0.15 g of multifloral spring honey from Lithuania and 250 µL of water was added to each vial with hydrated bee pollen. The honey was added as an additional carbohydrate source to support bacterial fermentation. Then for bacterial fermentation, the mixture was supplemented with 80 µL of the respective lactic acid bacteria culture, whereas 80 µL of MRS broth was added to samples intended for spontaneous fermentation. The vials were sealed and incubated at 37 °C to initiate fermentation for a proper time, as indicated in Table 2. Following addition of the hydration solution, honey solution, and bacterial inoculum, the pollen formed a thick, moist paste-like substrate without a free-flowing liquid phase.

### 2.4. Enzymatic Hydrolysis

For enzymatic hydrolysis, the following commercial enzymes were employed: Clara-diastase, Viscozyme L, and cellulase. Hydrolysis was conducted in 10 mL glass vials following the method described by Damulienė et al. [43] with minor modification.

Briefly, 1 g of each bee pollen sample was hydrated with 0.5 mL of sterile bidistilled water and then heated at 121 °C for 15 min. Each enzyme was applied at a dose of 0.05 U/g of bee pollen (calculated based on the activity units declared by the respective manufacturer for each commercial preparation) and dissolved in 300 µL of the appropriate buffer adjusted to the optimal pH prior to thorough mixing with the prepared samples. A 0.1 M sodium acetate buffer was used for pH 5.0, whereas a 0.1 M sodium phosphate buffer was used for pH 6.8. The hydrolysis conditions were as follows: Viscozyme L was applied at 40 °C and pH 5.0; cellulase at 37 °C and pH 5.0; Clara-diastase at 25 °C and pH 6.8. Mixtures containing a proper enzyme were incubated for 3 h 15 min. The hydrolysis process was terminated by immersing the vials in boiling water for 2 min.

### 2.5. Multi-Stage Bioprocessing

The multi-stage bioprocessing was performed by applying bacterial or spontaneous fermentation followed by enzymatic hydrolysis process or vice versa.

Treatment durations for the multi-stage bioprocesses were selected based on preliminary time-course optimization experiments using a representative bee pollen sample separately for each microorganism and multi-stage treatment sequence, with TPC, TFC, RSA, and inhibition of heat-induced bovine serum albumin denaturation (BSA-DI) activity monitored at different incubation times (2, 4, 8, and 12 days). The optimal durations for enzymatic hydrolysis were established in our previous study [43]. The optimized treatment durations used in the present study are summarized in Table 2.

In the first approach, bacterial fermentation was conducted as the initial bee pollen processing stage as described in Section 2.3, followed by enzymatic hydrolysis as described in Section 2.4. The second approach was carried out in reverse order, employing enzymatic hydrolysis as the first stage, followed by bacterial or spontaneous fermentation.

When enzymatic hydrolysis was applied as the first stage, the enzymes (Viscozyme L, Clara-diastase, and cellulase) were used at their respective optimal pH values (5.0, 6.8, and 5.0). Upon completion of hydrolysis, the samples were subjected to bacterial fermentation without any further pH adjustment.

When bacterial fermentation was performed as the first stage, no pH adjustment was applied during fermentation. The lactic acid bacteria were previously cultivated in MRS broth (pH 6.2). After completion of the fermentation stage, enzymatic hydrolysis was carried out without further modification of the pH.

All bioprocessing treatments were performed under their respective optimized conditions, including treatment duration, as summarized in Table 2. Therefore, treatment approaches were compared under their optimized conditions rather than at an identical incubation time.

### 2.6. Preparation of Extracts and Their Analysis

One gram of natural bee pollen and the entire treated material obtained from 1 g of initial bee pollen were subjected to extraction with 10 mL of 80% methanol. To ensure comparable sample handling, natural samples were pre-hydrated with 0.5 mL of distilled water and equilibrated for 1 h prior to extraction, whereas treated samples were extracted after the respective bioprocessing step. After 24 h of extraction at room temperature in an orbital shaker (LBX Orb-Pro, Labbox Labware S.L., Barcelona, Spain), the samples were filtered through a 7–10 μm paper filter, followed by further filtration using a 0.22 μm polyvinylidene fluoride (PVDF) membrane filter (BGB Analytik, Alexandria, VA, USA) for all spectrophotometric tests.

### 2.7. Spectrophotometric Determination of Bioactive Properties

The methods for determining total phenolic content (TPC), total flavonoid content (TFC), and radical scavenging activity (RSA) were performed according to Adaškevičiūtė et al. [44]. The reaction mixtures were prepared using an Inteliwasher 3D-IW8 microplate washer (Biosan Laboratories, Riga, Latvia) and measured at a specific wavelength using a Hipo MPP-96 microplate reader (Biosan Laboratories, Riga, Latvia).

Briefly, total phenolic content was determined using the Folin–Ciocalteu method as follows. Bee pollen extracts (8 μL) were mixed with 240 μL of 3.5% Na_2_CO_3_ and 8 μL of Folin–Ciocalteu reagent (The Merck Group, Darmstadt, Germany). The absorption was measured at a wavelength of 700 nm after incubation at 22 °C for 30 min. A calibration curve was constructed using rutin standards (0.1–1.0 mg/mL), and the regression equation was applied for quantification of total phenolic content in the samples. The results are expressed as mg rutin equivalents (RUE) per 1 g of initial raw bee pollen.

Total flavonoid content was determined using a colorimetric method. The stock solution comprised 60 mL of methanol, 3 mL of 33% acetic acid, 12 mL of 5% hexamethylenetetramine, 9 mL of 10% aluminum chloride, and 60 mL of bidistilled water. Each prepared bee pollen extract (10 μL) was mixed with 240 μL of the stock solution and incubated at 4 ± 2 °C for 30 min. After incubation, the samples were measured at a wavelength of 407 nm. A calibration curve was constructed using rutin solutions (0.1–1.0 mg/mL), and the obtained regression equation was used to calculate the total flavonoid content in the samples. The results are expressed as mg rutin equivalents (RUE) per 1 g of initial raw bee pollen.

Radical scavenging activity was determined using a colorimetric assay based on the 1,1-diphenyl-2-picrylhydrazyl (DPPH) free radical. Bee pollen extracts (5.9 μL) were mixed with 230 μL of the radical stock solution, composed of 0.1 M acetate buffer (pH 5.5), acetonitrile, and methanol (1:1.25:1.25), with an initial absorbance of 0.500 at 515 nm. The prepared samples were incubated at 22 °C in the dark for 15 min and then measured at the wavelength of 515 nm. Radical scavenging activity was determined based on a rutin calibration curve (0.1–0.25 mg/mL). The results are expressed as mg rutin equivalents (RUE) per 1 g of initial raw bee pollen.

In vitro inhibition of heat-induced bovine serum albumin denaturation assay (BSA denaturation inhibition assay—BSA-DI), used as a preliminary in vitro indicator of potential anti-inflammatory activity, was evaluated using the method described by Mizushima et al. [45] with slight modifications by Sakat et al. [46]. A 1% aqueous solution of bovine serum albumin was prepared, and phosphate-buffered saline (PBS) buffer was adjusted to pH 6.4. The reaction mixture consisted of 50 µL of sample, 450 µL of 1% bovine serum albumin solution, and 2.5 mL of PBS buffer. The samples were incubated at 37 °C for 20 min to allow interaction between the sample and bovine serum albumin, followed by heating at 57 °C for 20 min to induce protein aggregation, and then immediately cooled on ice. After incubation and cooling, the reaction mixtures were transferred to a 96-well microplate, and absorbance was measured at 660 nm using a microplate reader. A reference solution was prepared in the same manner using distilled water instead of the sample. For calibration, salicylic acid standard solutions (0.1–15.0 mg/mL) were prepared, and results were expressed as salicylic acid equivalents (SAE) per gram of initial raw bee pollen [45,46].

All spectrophotometric assays, including TPC, TFC, RSA, and BSA-DI, were performed in ten technical replicates for each prepared extract. The mean of the technical replicates was used for subsequent statistical analysis.

### 2.8. Chemometric Analysis

Data mining of obtained results was carried out in MATLAB R2025b software (MathWorks, Inc., 2025, Natick, MA, USA). The aims of the statistical data mining were as follows: (1) to determine whether multi-stage bioprocessing results in a statistically significant increase in BSA-DI, TPC, TFC, and RSA; (2) to identify which combination of multi-stage bioprocessing yields the best results; and (3) to determine the clusters (groups) formed by the applied multi-stage bioprocessing processes based on the similarity of the measured characteristics. Statistical analysis included hypotheses testing applying Student’s *t*-test, correlation analysis evaluating Pearson correlation coefficient, analysis of variance and hierarchical cluster analysis [47].

Additional data preprocessing was performed where required by statistical analysis. Depending on the specific analysis, preprocessing involved averaging repeated measurements or averaging repeated measurements across samples, followed by normalization according to Equations (1) or (2) and Equation (3), respectively:

(1) $${\bar{x}}_{kj}=\frac{1}{M}\sum_{i=1}^{M}x_{kji},$$

(2) $${\bar{x}}_{k}=\frac{1}{N}\frac{1}{M}\sum_{j=1}^{N}\sum_{i=1}^{M}x_{kji},$$

(3) $$z=\frac{\bar{x}-min(\bar{x})}{\max\left(\bar{x}\right)-min(\bar{x})},$$

where *k*, *j* and *i* denote the indices of the applied pollen treatments, samples, and measurements, respectively, *M* and *N* represent the total number of measurements and samples, respectively, $\bar{x}$ represents an averaged data matrix, obtained after applying (1) or averaged data vector, obtained after applying (2).

To present changes in the magnitude of the measured TPC, TFC, RSA, and BSA-DI following the application of various fermentation treatments, the data were visualized using heatmaps. Prior to visualization, the data were averaged using (2) and subsequently normalized using (3).

A one-tailed (right-tailed) Student’s *t*-test was employed to assess pairwise differences between the mean values of the measured TPC, TFC, RSA, and BSA-DI for individual samples or sample groups, specifically to test the hypothesis that bioprocessing resulted in a statistically significant increase in the measured characteristics, at a significance level of *p* < 0.05. The hypothesis-testing analysis was performed using preprocessed measurands obtained by averaging the repeated measurements for each measurand and subsequently applying normalization, as described above (Equations (1) and (3)). Because multiple pairwise *t*-tests were performed, the resulting *p*-values were adjusted for multiple hypothesis testing using the Bonferroni correction to control the family-wise error rate. Statistical significance was finally determined based on the Bonferroni-adjusted *p*-values.

Subsequently, Pearson correlation analysis was performed using averaged and normalized data using Equations (1) and (3), respectively, to reveal the relationships among TPC, TFC, RSA, and BSA-DI. This analysis served as the basis for deriving a composite variable calculated as a weighted sum of the measured characteristics.

To assess the distinctiveness of the investigated bee pollen samples from different regions of Lithuania based on the measured characteristics, analysis of variance (ANOVA) was applied. Multiple pairwise comparisons were performed to identify which sample means differed significantly. The Tukey–Kramer multiple-comparison procedure was applied for these post hoc comparisons, thereby accounting for the multiplicity of pairwise comparisons and controlling the family-wise error rate. Statistical significance of the pairwise differences was assessed using the Tukey–Kramer adjusted *p*-values at a significance level of *p* < 0.05.

Hierarchical cluster analysis (HCA) was employed to reveal the similarities among the applied fermentations, allowing them to be grouped into distinct clusters. Euclidean distance was used as the similarity measure, coupled with Ward’s linkage method, which minimizes the total within-cluster variance at each step of cluster formation. The clustering results were presented as dendrograms, which were cut to define clusters at a level corresponding to 0.5 of the maximum Euclidean distance. As no universally accepted criterion exists for selecting the optimal dendrogram cut-off level, this threshold was adopted as a practical heuristic to facilitate cluster interpretation. To assess the reliability of the hierarchical clustering, the cophenetic correlation coefficient was calculated to evaluate the agreement between the original pairwise distances and the cophenetic distances represented by the dendrogram.

## 3. Results and Discussions

### 3.1. Impact of the Treatment Method on Bee Pollen Activity

Previous studies have demonstrated that both bacterial fermentation and enzymatic hydrolysis enhance the release of biologically active compounds from bee pollen [29,42,43] and may improve its functional properties, including anti-inflammatory activity [48,49,50] and cardiovascular health-promoting properties [51]. However, the efficiency of these bioprocesses strongly depends on several factors, including the microorganism species and strain, enzyme type, processing duration, and the origin of the bee pollen.

Although bacterial fermentation is an effective strategy for improving the bioaccessibility of pollen constituents, the process is often relatively time-consuming. Prolonged fermentation may also increase the risk of contamination by undesirable microorganisms, including potentially pathogenic species [52,53]. Bee pollen may also contain mycotoxin-producing fungi, and the occurrence of fungal contamination and mycotoxins can be influenced by processing and storage conditions [21,22]. Microbiological safety should be considered when developing multi-day fermentation processes for practical applications. In the present study, microbial dynamics, contamination, and mycotoxin formation were not assessed; therefore, further studies are required to validate the microbiological safety and stability of the proposed bioprocessing approach before its application in food or nutraceutical products.

**Figure 2:**
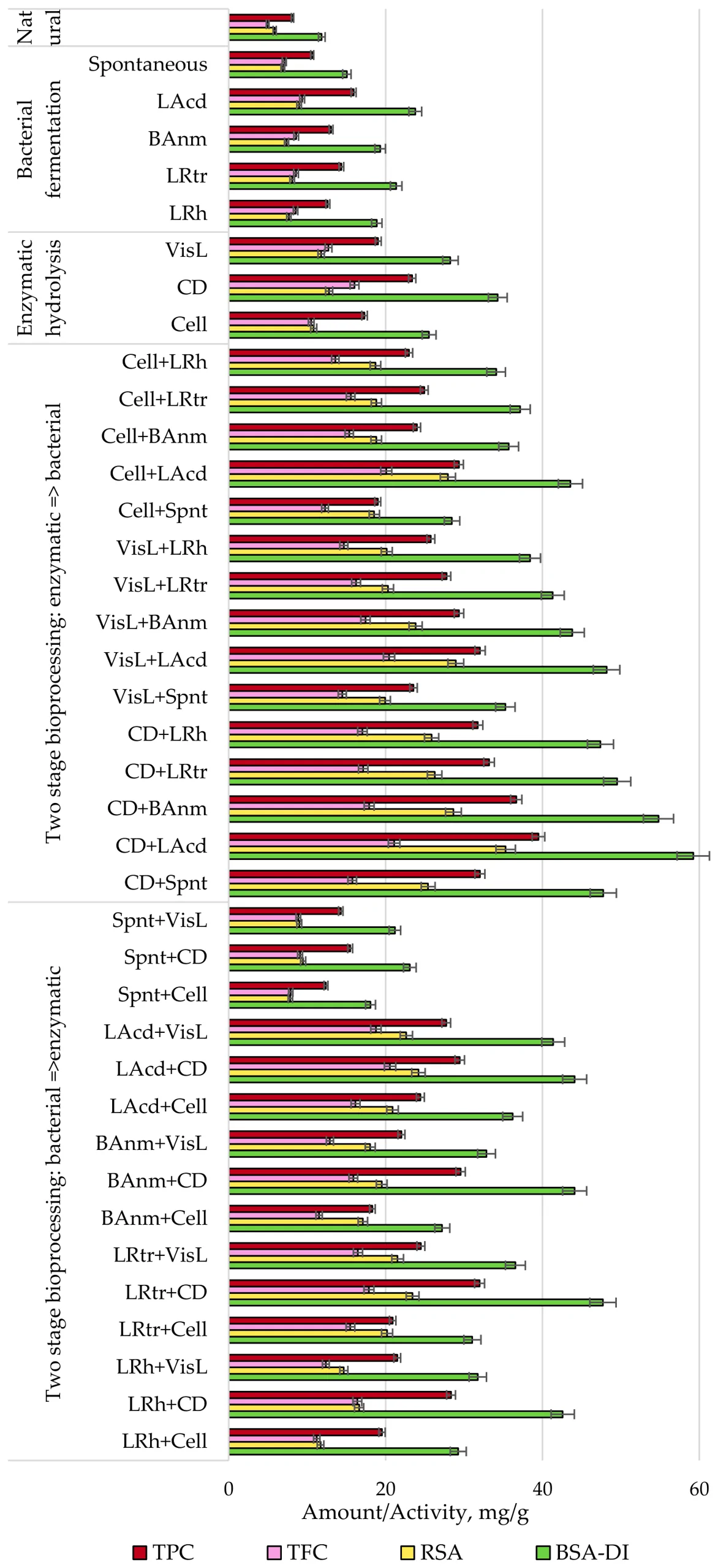
Effect of single and hybrid bioprocessing treatments on total phenolic content (TPC), total flavonoid content (TFC), radical scavenging activity (RSA), and BSA denaturation inhibition (BSA-DI) of a bee pollen sample collected in Marijampolė County (10 technical replicates; RSD ≤ 3.5%). Treatment abbreviations are given in Table 2. All values differed significantly from those of natural bee pollen (*p* < 0.05).

Combining enzymatic hydrolysis and fermentation in a sequential manner could represent an alternative strategy for enhancing the recovery of bioactive compounds within a shorter incubation period. Accordingly, the aim of this study was not only to increase the content of biologically active compounds and improve the bioactivity-related properties of bee pollen extracts but also to evaluate whether multi-stage bioprocessing could achieve these improvements within shorter incubation periods than single-stage bacterial fermentation. To the best of our knowledge, this is the first study investigating such a hybrid bioprocessing strategy for bee pollen using both possible sequences of enzymatic hydrolysis and fermentation. Figure 2 and Table 3 represent changes in total phenolic content (TPC) and total flavonoid content (TFC), also variation in radical scavenging activity (RSA) and BSA denaturation inhibition (BSA-DI) in one of the tested samples. These results indicate that all treatment methods positively influenced the recovery of bioactive compounds and their associated activities.

As shown in Figure 2 and Table 3, the treatment method had a significant effect on both the content of bioactive compounds and the biological activity of the extracts. Prior to treatment, TPC in bee pollen samples ranged from 5.8 to 13.3 mg/g RUE.

The smallest changes in TPC were observed following spontaneous fermentation; however, these increases remained statistically significant (*p* < 0.05), ranging from 1.1 to 1.3-fold. Bacterial fermentation resulted in a more pronounced increase in TPC (1.6–2.0-fold), whereas the combination of spontaneous fermentation and enzymatic hydrolysis produced intermediate increases (1.4–1.9-fold). The highest TPC value was obtained after multi-stage bioprocessing (40.1 mg/g RUE), whereas the maximum value achieved after single treatments reached only 26.2 mg/g RUE. Similarly, the minimum TPC value after multi-stage processing (13.5 mg/g RUE) was comparable to that observed following single treatments (14.4 mg/g RUE).

A similar trend was observed for TFC. Natural bee pollen samples contained 3.7–8.4 mg/g RUE of flavonoids, and spontaneous fermentation increased TFC by only 1.3–1.4-fold. In contrast, bacterial fermentation resulted in a 1.6–1.9-fold increase, while combined spontaneous fermentation and enzymatic hydrolysis led to a 1.5–1.8-fold increase. Multi-stage bioprocessing resulted in higher TFC values than single treatments, with maximum and minimum values of 24.1 and 8.9 mg/g RUE, respectively, compared to 18.0 and 9.5 mg/g RUE after individual treatments.

Prior to treatment, RSA ranged from 4.3 to 9.9 mg/g RUE. Spontaneous fermentation resulted in a consistent 1.2-fold increase across all samples. Bacterial fermentation increased RSA by 1.2–1.6-fold, whereas the combined spontaneous fermentation and enzymatic hydrolysis treatment led to a 1.3–1.7-fold increase. Compared with the most effective fermentation and hybrid treatments, cellulase hydrolysis produced a more moderate yet statistically significant (*p* < 0.05) increase in RSA (1.6–1.8-fold). The superiority of multi-stage bioprocessing was particularly evident for RSA, where the maximum activity reached 35.2 mg/g RUE, compared with 14.0 mg/g RUE obtained after single treatments. The minimum RSA values were similar for both treatment approaches (9.0 and 8.8 mg/g RUE, respectively).

The BSA-DI of untreated bee pollen samples ranged from 8.7 to 18.8 mg/g SAE. Similar to TPC and TFC, the smallest increases were observed after spontaneous fermentation (1.1–1.3-fold). Bacterial fermentation resulted in a 1.6–2.1-fold increase in BSA-DI, while combined spontaneous fermentation and enzymatic hydrolysis produced intermediate increases ranging from 1.4 to 2.0-fold. The highest BSA-DI was recorded after multi-stage bioprocessing (59.6 mg/g SAE), substantially exceeding the maximum value observed after single treatments (38.6 mg/g SAE). Likewise, the minimum activity after multi-stage processing (19.5 mg/g SAE) remained comparable to that obtained following individual treatments (21.1 mg/g SAE). Previous studies have shown that natural bee pollen exhibits anti-inflammatory activity linked to its polyphenolic and flavonoid content, influencing inflammatory pathways and enzyme activity [54,55,56]. However, limited information is available regarding the effects of fermentation and enzymatic hydrolysis on its BSA-DI potential.

The relatively modest increases observed after spontaneous fermentation suggest that fermentation by the native microbial communities present in bee pollen may have a more limited effect on the recovery of bioactive compounds under the conditions applied in the present study. Spontaneous fermentation is a naturally occurring process that takes place when environmental conditions are favourable, initiated by native microbial communities present in the pollen. These microorganisms typically include bacterial genera such as *Pseudomonas* and *Lactobacillus*, along with yeast species like *Saccharomyces* [25]. In contrast, bacterial fermentation using selected lactic acid bacteria (*L. rhamnosus*, *L. reuteri*, *B. animalis*, *L. acidophilus*) resulted in a more pronounced enhancement of all measured parameters, which may be attributed to the specific metabolic and enzymatic activities of the applied strains, potentially facilitating the release of bound phenolic compounds and improving their extractability. *L. acidophilus* fermentation demonstrated the highest impact on the measured bioactive compounds contents and activities of biologically active compounds across the samples, which may be associated with its reported carbohydrate metabolism and glycosidase activity, potentially facilitating the release of compounds from the pollen matrix and phenolic glycosides and increasing their bioaccessibility [57]. However, the endogenous microbiota of the pollen samples was not characterized in the present study; therefore, its specific contribution to the observed effects cannot be determined.

Enzymatic hydrolysis may have facilitated the release and recovery of phenolic and flavonoid compounds [29,30]. When combined with fermentation, either in sequential or reverse order, intermediate effects were observed, suggesting a synergistic or partially overlapping action of microbial and enzymatic processes. These effects may be associated with changes in the pollen matrix and microbial metabolism, although the specific structural changes were not directly examined in the present study.

**Figure 3:**
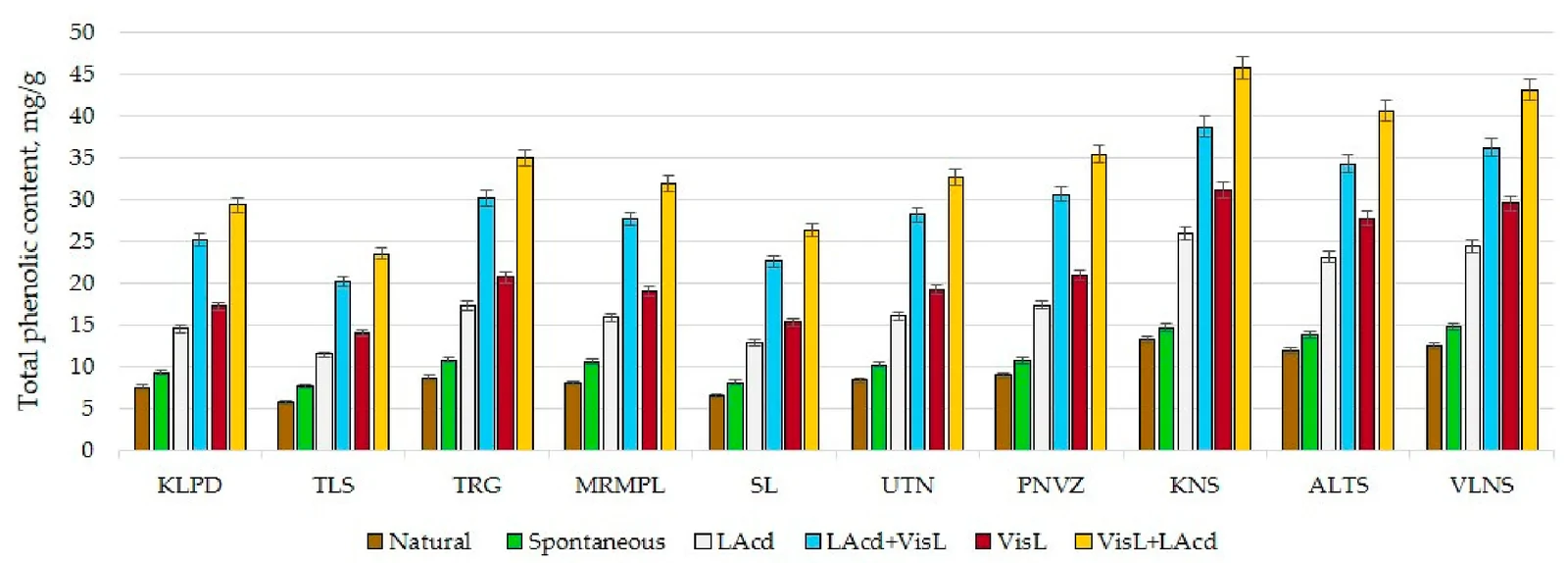
Changes in total phenolic content (TPC, mg/g RUE) in bee pollen during spontaneous fermentation, single *Lactobacillus acidophilus* fermentation, single Viscozyme L hydrolysis, and their combined treatments (10 technical replicates; RSD ≤ 3.5%; abbreviations are given in Table 2 and Table 3). All values differed significantly from those of natural bee pollen (*p* < 0.05).

The highest effects, as expected, were observed after combined bioprocessing treatments. Compared with the individual natural bee pollen samples, TPC increased significantly by 3.7–5.0-fold, TFC by 4.0–4.2-fold, RSA by 4.9–6.3-fold, and BSA-DI by 3.7–5.3-fold, depending on the sample and treatment combination. It is difficult to identify a single superior treatment, as the response was influenced by the individual bee pollen sample (Figure 3, Tables S1–S4). Nevertheless, the greatest increases were most frequently observed when Clara-diastase was combined with bacterial fermentation, irrespective of whether hydrolysis preceded fermentation or vice versa.

Notably, the Clara-diastase treatment also demonstrated promising results when combined with spontaneous fermentation, yielding 3.0–4.6-fold increases across the analysed samples. The pronounced effect of this sequential treatment may be associated with enhanced accessibility and recovery of phenolic and other bioactive compounds following enzymatic treatment. Since the pollen samples were autoclaved prior to hydrolysis, microbial activity originating from the native pollen microbiota is unlikely to be the primary mechanism underlying the observed changes. Instead, the continued release of bioactive compounds from the pollen matrix and, potentially, residual enzymatic activity may have contributed to the observed increases in TPC, TFC, RSA, and BSA-DI. However, the specific structural changes induced by enzymatic hydrolysis were not directly assessed in the present study.

The observed results suggest that the combination of fermentation and enzymatic hydrolysis can significantly enhance the antioxidant properties of bee pollen. To the best of our knowledge, the sequential application of spontaneous or bacterial fermentation and enzymatic hydrolysis in both possible orders has not been systematically investigated for bee pollen with a focus on enhancing the recovery of bioactive compounds and related in vitro activities. Sequential hydrolysis and fermentation have been widely applied in the treatment of agro-industrial by-products for the recovery of functional ingredients [58,59] and as a pretreatment strategy for bioethanol production [60,61]. In the case of bee pollen, previous studies have mainly focused on fermentation using single strains or mixed microbial cultures [58,62,63], rather than combining fermentation and enzymatic hydrolysis within a multi-stage bioprocessing approach.

Plausible, enzymatic hydrolysis followed by bacterial fermentation enhances the activity of bee pollen by increasing the availability of low-molecular-weight bioactive compounds prior to microbial action. Hydrolysis breaks down complex structures into smaller peptides, amino acids, and phenolic-related compounds, generating a substrate more accessible to microbial metabolism [64]. This facilitates selective microbial transformation and may preserve or further enhance biologically active molecules formed during hydrolysis [33,63,64].

### 3.2. Chemometric Evaluation of Bioprocessing Treatments

Bee pollen samples (10 in total), collected from different regions of Lithuania, were analysed in this study following the application of single- and multi-stage bioprocessing. As presented above (Table 2), 8 single-stage and 30 multi-stage fermentation treatments were applied across all samples. Total phenolic compound content, total flavonoid content, radical scavenging activity, and inhibition of heat-induced BSA denaturation were each measured 10 times for every pollen sample in its raw form (natural bee pollen, used as the control) as well as after fermentation under 38 described different conditions. In total, a dataset of size of [390 × 10] comprising 39 treatments applied to each of the 10 bee pollen samples, with 10 repeated measurements per treatment, was generated for TPC, TFC, RSA, and BSA-DI.

Following the statistical analysis plan described in Section 2.8, one-tailed (right-tailed) Student’s *t*-tests were performed to evaluate whether bioprocessing resulted in statistically significant increases in TPC, TFC, RSA, and BSA-DI. The tested hypotheses were: (1) the mean values of the measurands after single-stage bioprocessing are greater than those of natural bee pollen (control); (2) the mean values of the measurands after multi-stage bioprocessing are greater than those of natural bee pollen (control); and (3) the mean values of the measurands after multi-stage bioprocessing are greater than those after single-stage bioprocessing.

**Figure 4:**
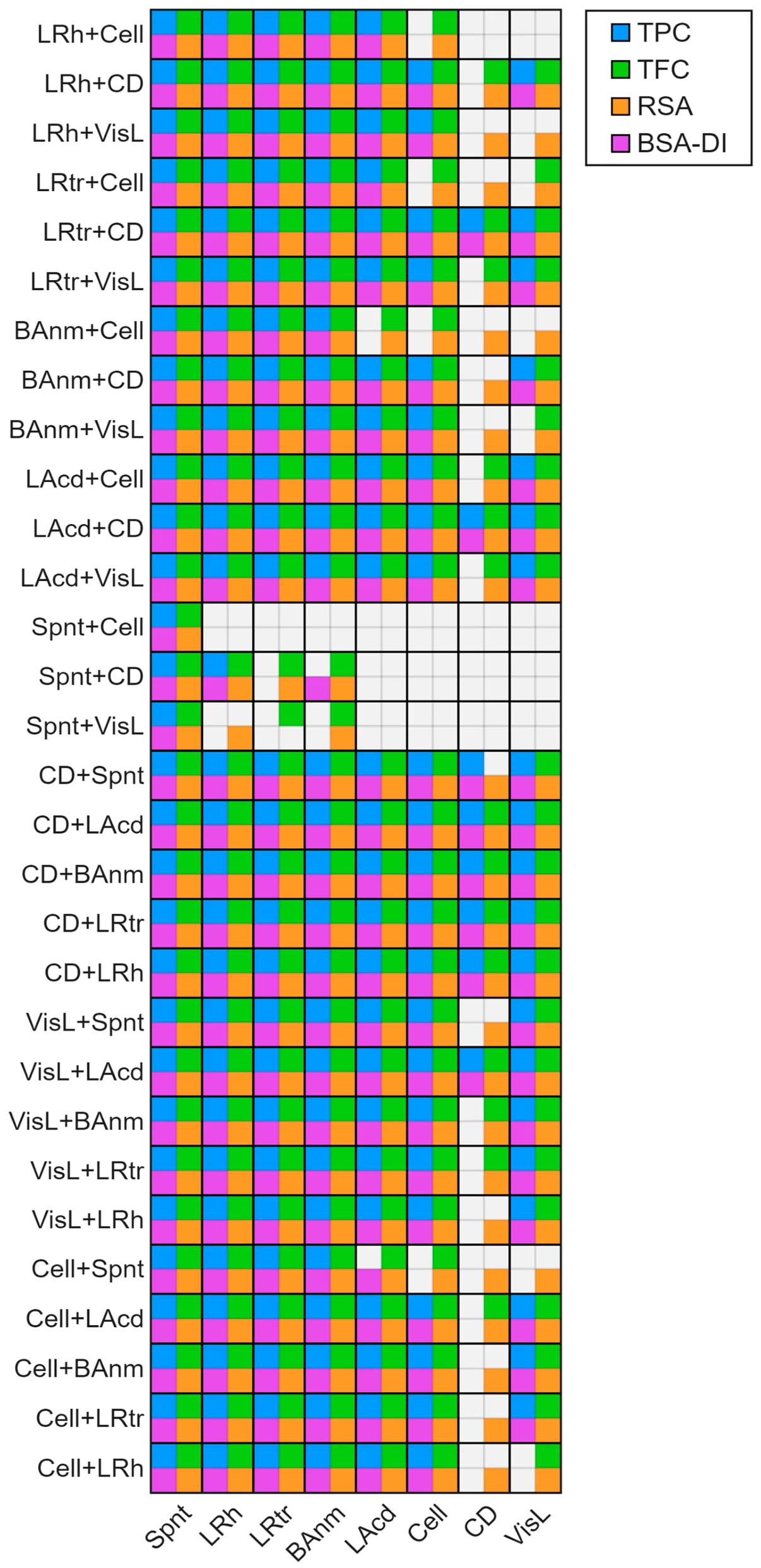
Student’s *t*-test comparison of increases in total flavonoid content (TFC), radical scavenging activity (RSA), and inhibition of heat-induced bovine serum albumin denaturation (BSA-DI) following multi-stage versus single-stage bioprocessing at a significance level of *p* < 0.05. Statistically significant increases with multi-stage bioprocessing are indicated by solid colors. Treatment abbreviations are given in Table 2.

The first and second groups of directional hypotheses remained statistically significant after application of the Bonferroni correction at a significance level of 0.05, confirming that both single-stage and multi-stage bioprocessing produced significant increases in TPC, TFC, RSA, and BSA-DI compared with the untreated control samples. In contrast, the comparison between single-stage and multi-stage bioprocessing revealed that not all multi-stage treatment combinations outperformed their single-stage counterparts. In particular, multi-stage processes employing spontaneous fermentation as the first processing stage exhibited lower overall performance than most single-stage bioprocessing approaches using bacteria or enzymes. A similar trend was observed for treatment with the enzyme Clara-Diastase, which, when applied as a single-stage process, yielded significantly greater improvements of the measured characteristics than most of the investigated multi-stage bioprocessing combinations. These findings indicate that the incorporation of an additional processing stage strongly depends on the specific combination and sequence of the applied treatments. Figure 4 presents the detailed results of the discussed third group of hypothesis tests.

The subsequent correlation analysis revealed very strong correlations among all measured characteristics (Figure S1). The lowest Pearson correlation coefficient was observed between RSA and BSA-DI (0.910, at level *p* < 0.05), while the highest was found between TPC and BSA-DI (0.990, at level *p* < 0.05). A composite variable, weighted sum (WS), was derived to provide an integrated representation of the four measured characteristics: TPC, TFC, RSA, and BSA-DI. WS was constructed based on the relationships among these characteristics, as assessed by correlation analysis. As the correlation coefficients were highly similar in magnitude, no individual characteristic showed a substantially different contribution to the common response pattern. Therefore, equal weights were assigned to the four components in the WS calculation. The WS was used as an integrated comparative indicator of treatment effects, while the individual characteristics were retained for separate statistical analysis and interpretation.

**Figure 5:**
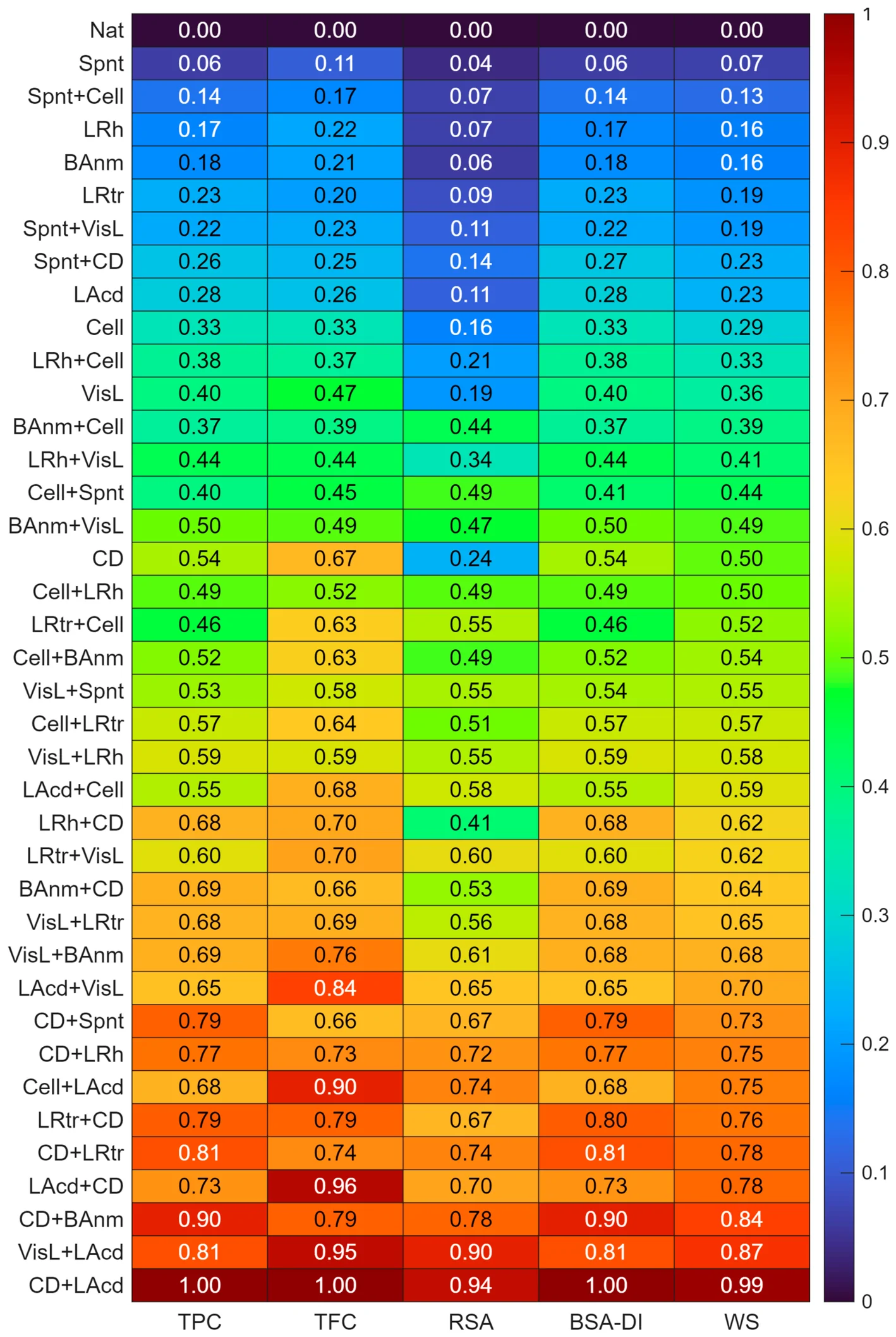
Heatmap of measured total phenolic content (TPC), total flavonoid content (TFC), radical scavenging activity (RSA), and inhibition of heat-induced bovine serum albumin denaturation (BSA-DI), together with derived weighted sum (WS). Treatment abbreviations are given in Table 2.

The heatmap diagram of averaged, normalized and ordered in ascending order according to WS, is presented in Figure 5. The heatmap representing the incremental normalized changes in the measured characteristics of the pollen samples, confirmed a clear positive effect of multi-stage bioprocessing on the yield of bioactive compounds. The visualization indicated the presence of at least three distinct groups of treatments, namely low-, medium-, and high-yield groups.

The low-yield group comprised natural bee pollen and primarily single-stage bioprocessing, as well as multi-stage bioprocessing in which spontaneous fermentation was applied as the initial step. In contrast, the high-yield group consisted predominantly of multi-stage bioprocessing processes, particularly those involving Clara-diastase, Viscozyme L, and cellulase in at least one stage. The most effective combinations were observed when these enzymes were applied during the initial stage of bioprocessing. This finding suggests that the initial enzymatic may have contributed substantially to the observed changes in the pollen biochemical profile, potentially by facilitating the disruption of the pollen matrix and increasing the extractability of bioactive compounds. However, the structural changes underlying these effects were not directly investigated in the present study. Notably, the use of these enzymes in single-stage bioprocessing did not result in comparably high yields. Among all tested multi-stage bioprocessing treatments of bee pollen, the optimal treatment was the combination of Clara-diastase and *L. acidophilus*.

The medium-yield group represented an intermediate cluster of fermentation treatments, characterized by moderate increases in all measured bioactive parameters. Compared to the low-yield group, the medium-yield treatments demonstrated a clear improvement in bioactive compound extraction, revealing that the introduction of additional bioprocessing steps enhances overall efficiency. However, their performance remained consistently below that of the high-yield group, indicating that not only the number of stages but also the specific combination and sequence of enzymes and/or bacterial strains played a critical role.

**Figure 6:**
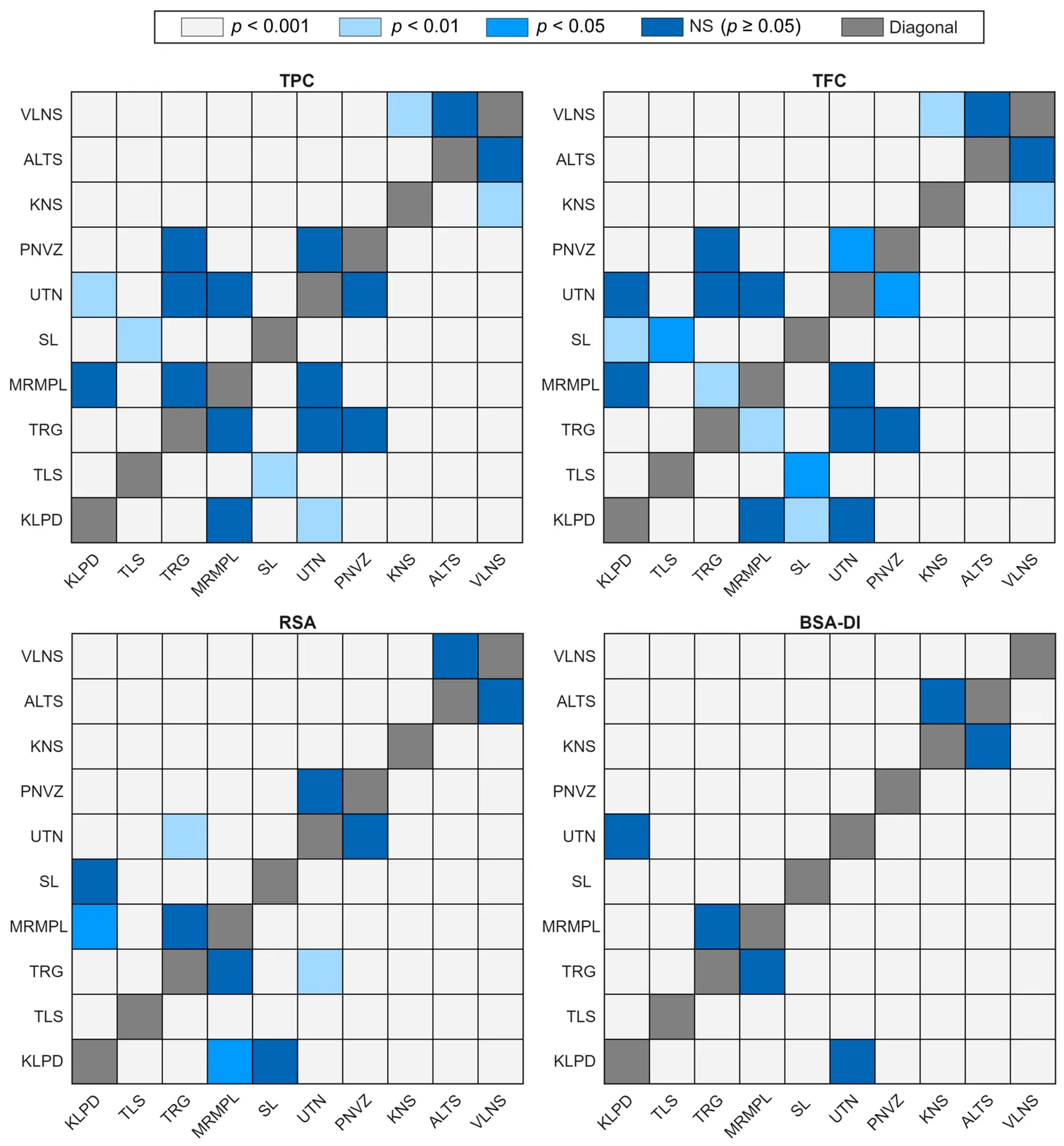
ANOVA results of tested samples pair-wise comparison based on the measured total phenolic content (TPC), total flavonoid content (TFC), radical scavenging activity (RSA), and inhibition of heat-induced bovine serum albumin denaturation (BSA-DI). NS—no significant difference observed. Samples abbreviations are given in Table 1.

ANOVA was applied to assess and reveal the distinctiveness of investigated bee pollen samples from various regions of Lithuania based on the measured characteristics. This analysis was conducted on natural bee pollen samples prior to any treatment to characterize the variability of the starting materials used in the subsequent multi-stage bioprocessing experiments. Figure 6 illustrates the ANOVA results for TPC, TFC, RSA, and BSA-DI, respectively.

The rationale for this analysis was to assess whether the collected sample set encompassed sufficient variability in the measured characteristics to provide a heterogeneous set for evaluating and generalizing the effects of the bioprocessing treatments. If the samples were excessively similar, the range of pollen characteristics would be limited, potentially restricting the applicability of the observed treatment effects to a specific type of pollen. Conversely, the presence of appreciable variability among the pollen samples provides a broader basis for evaluating the robustness of the treatment effects.

The ANOVA results revealed that the samples differed significantly, although statistically significant differences were not observed for all comparisons indicating similar bioactivity profiles. For example, some geographically proximate sample pairs, including VLNS (Vilnius)–ALTS (Alytus), MRMPL (Marijampolė)–TRG (Tauragė), and PNVZ (Panevėžys)–UTN (Utena), did not exhibit statistically significant differences for the investigated characteristics. Overall, the observed variation among the ten samples indicated that the experimental set encompassed a range of initial pollen characteristics suitable for evaluating the effects of the subsequent multi-stage bioprocessing treatments.

**Figure 7:**
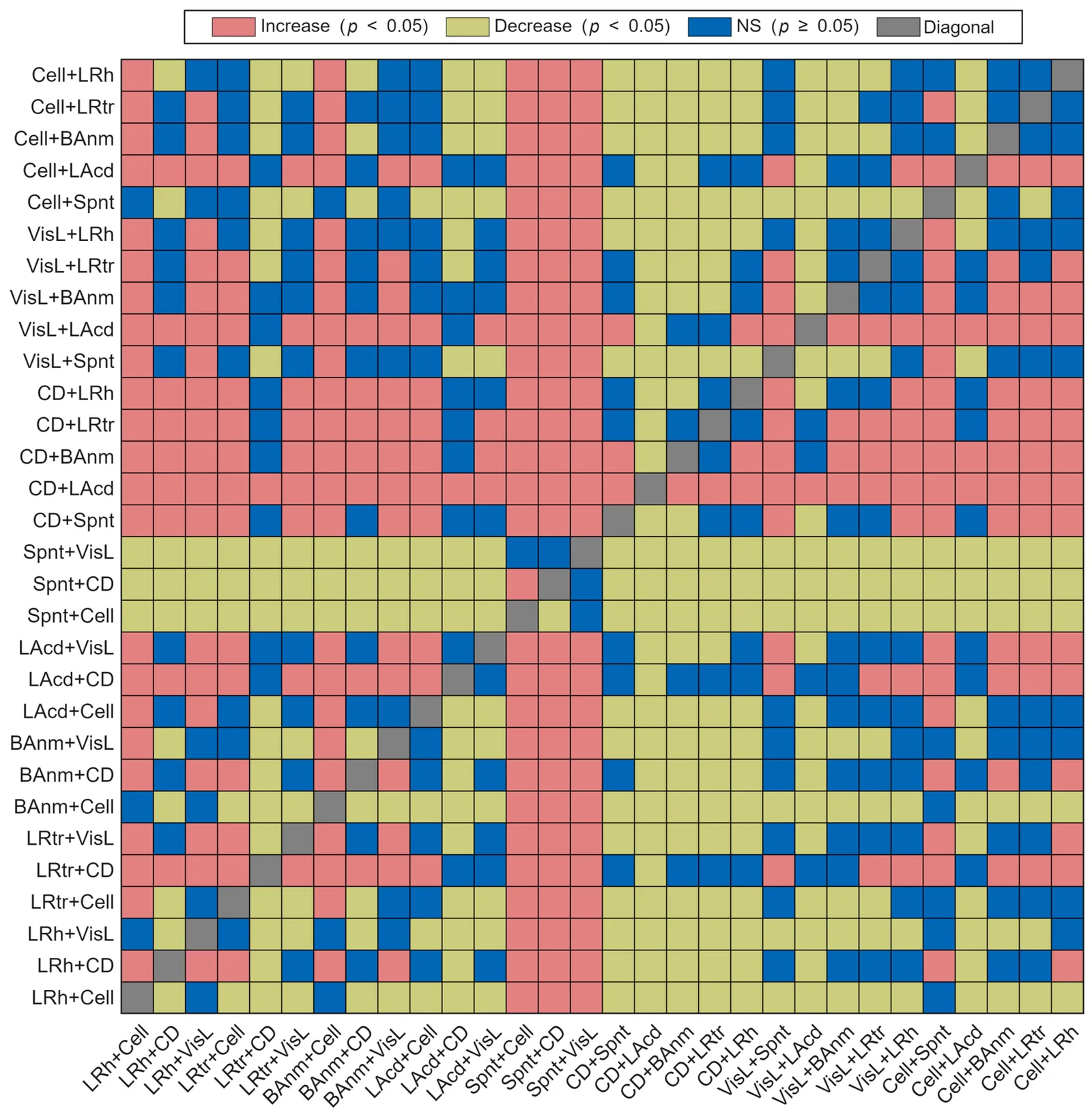
ANOVA results of multi-stage bioprocessing pair-wise comparison based on the weighted sum (WS) of measured total phenolic content (TPC), total flavonoid content (TFC), radical scavenging activity (RSA), and inhibition of heat-induced bovine serum albumin denaturation (BSA-DI). Statistically significant increases or decreases refer to the bioprocesses on the vertical axis relative to the corresponding bioprocesses on the horizontal axis. NS—no significant difference observed. Treatment abbreviations are given in Table 2.

Following the evaluation of the samples, the same analysis methodology was applied to the fermentation treatments investigated in this study. The aim was to determine whether statistically significant differences existed among the treatments, given the rapidly increasing number of possible multi-stage bioprocessing strategies, arising not only from the variety of enzymes and bacterial strains used but also from the sequence of their application. Only multi-stage bioprocessing was included in this analysis, as this approach had previously been shown to provide better results than single-stage processes. Figure 7 presents the ANOVA results of pairwise comparisons of the mean values of the derived WS variable, illustrating the outcomes of hypotheses testing for equality and the identification of statistically significant differences among the applied treatments.

The presented Figure 7 illustrates that a number of combinations of enzymes and bacterial strains yielded similar results, with no statistically significant differences observed. Notably, all combinations involving spontaneous fermentation as the initial step exhibited statistically significant outcomes that differed from all other tested treatments. This suggests that the microflora introduced during spontaneous fermentation, originating from bee saliva, may be distinct from that associated with the bacterial strains applied in this study. However, these combinations generally resulted in lower yields of the measured characteristics. The most favourable results were observed when the Clara-diastase enzyme was applied during the initial pollen treatment step, producing significantly distinct outcomes from all other treatments, when this enzyme was not involved. In conclusion, as shown in Figure 5, the highest yields were obtained from multi-stage bioprocessing combinations that included Clara-diastase, Viscozyme L, and Cellulase. ANOVA analysis confirmed that this trend was consistent, demonstrating that multi-stage bee pollen treatment incorporating these enzymes yields reproducible and distinct outcomes, especially when applied in the initial single-stage.

**Figure 8:**
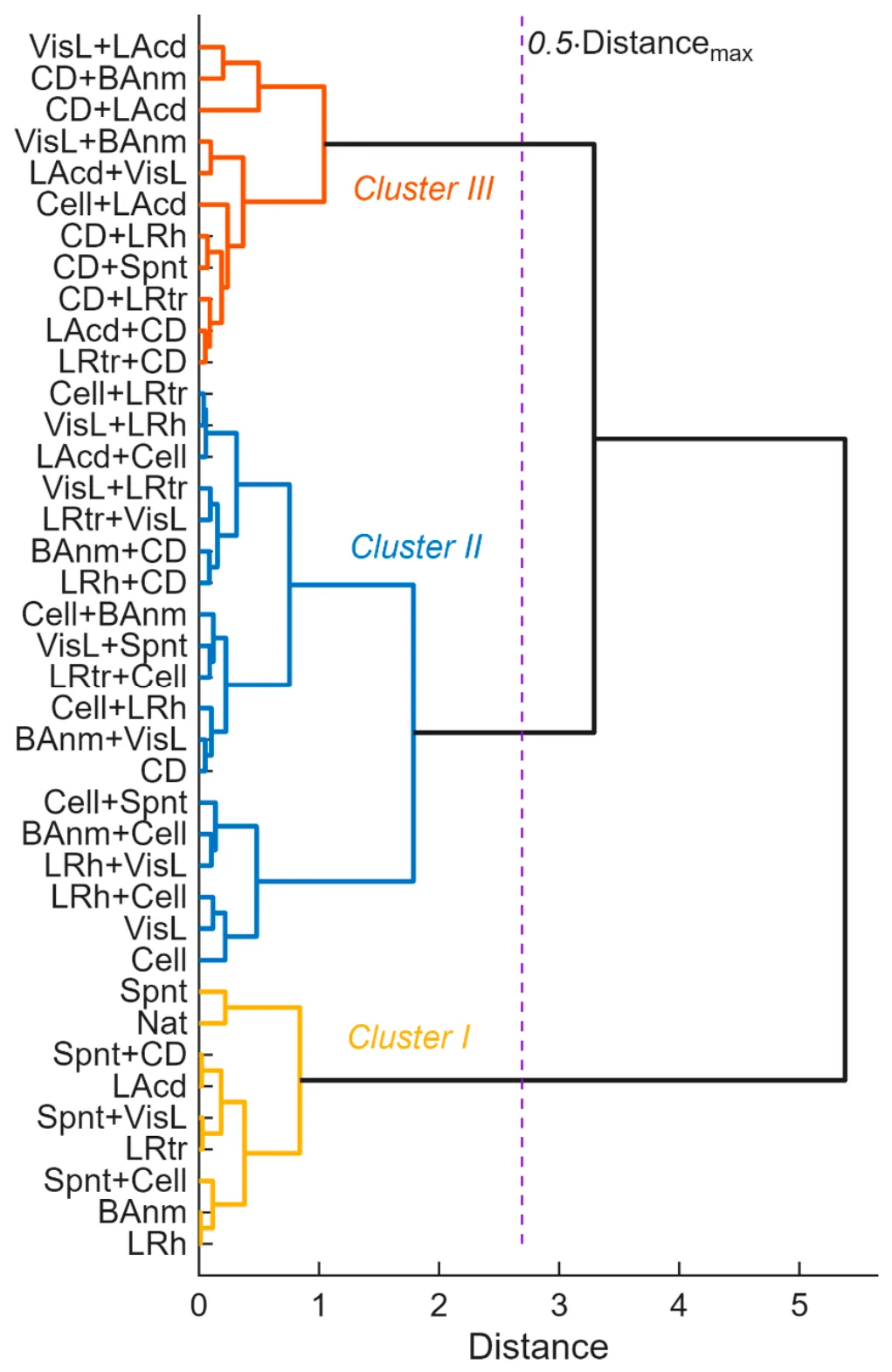
HCA clustering of treatments applied to bee pollen using weighted sum WS variable. Treatment abbreviations are given in Table 2.

To reveal the similarities among the applied bioprocessing approaches and to classify them according to their overall bioactivity, hierarchical cluster analysis (HCA) was performed using the averaged weighted-sum (WS) variable as the input. The WS variable served as an integrated indicator combining the four measured characteristics (TPC, TFC, RSA, and BSA-DI), thereby enabling comparison of the overall treatment performance. The resulting dendrogram is presented in Figure 8. The cophenetic correlation coefficient of the hierarchical clustering was 0.74, indicating a good correspondence between the original pairwise distances and the dendrogram representation and supporting the reliability of the identified cluster structure. The dendrograms of TPC, TFC, RSA and BSA-DI are presented in Figures S2–S5, respectively.

As anticipated from the heatmap (see Figure 5), hierarchical cluster analysis revealed three distinct clusters of pollen treatments. The first cluster comprised natural pollen, spontaneous fermentation and its combinations, as well as single-stage bacterial fermentations. Notably, this group did not include enzyme-based treatments, except for combinations involving spontaneous fermentation, which did not demonstrate significant improvements.

The second cluster consisted primarily of multi-stage bioprocessing in which bacterial treatment was applied as the initial stage. This group also included single-stage hydrolysis using Clara-diastase, Viscozyme L, and cellulase, as previously noted, suggesting that enzymatic hydrolysis may be more effective than bacterial fermentation under the tested conditions. This trend was especially pronounced in the third (high-yield) cluster, which comprised the most effective enzyme combinations, followed by bacterial fermentation in the second stage, enhancing the yield of measured TPC, TFC, RSA, and BSA-DI. The only exception to this trend was the *L. acidophilus* bacterial strain, which fell into high-yield cluster (cluster III) and exhibited measured characteristics comparable to those of the enzymatic treatments.

### 3.3. Study Limitations

Several limitations of the present study should be acknowledged. The evaluation was based on total phenolic content (TPC), total flavonoid content (TFC), radical scavenging activity (RSA), and inhibition of heat-induced bovine serum albumin denaturation (BSA-DI). Although these assays are useful for comparing bioprocessing treatments, DPPH and BSA-DI provide preliminary in vitro assessments and do not fully reflect biological responses in vivo. Further studies using appropriate in vivo models would be valuable for validating these findings.

The botanical origin of the pollen samples was not determined by palynological analysis; therefore, differences in floral composition may have contributed to variability among samples. The findings should consequently be interpreted in relation to the tested pollen samples and their geographic origin rather than specific botanical sources.

Fermentation kinetics were not monitored in detail, and changes in pH, viable-cell numbers, microbial dynamics, and metabolite production were not determined. Because treatment durations were optimized separately for each bioprocess and therefore differed between single-stage and multi-stage treatments, the contribution of treatment type relative to total incubation time cannot be fully separated. Further studies should address these aspects, together with microbiological safety, process mass balance, economic feasibility, and scalability, to support evaluation of the mechanisms and practical applicability of the proposed bioprocessing approach.

## 4. Conclusions

The study describes proposed multi-stage bioprocessing approach involving bacterial fermentation, spontaneous fermentation, and enzymatic hydrolysis to evaluate changes in total phenolic content (TPC), total flavonoid content (TFC), radical scavenging activity (RSA), and heat-induced bovine serum albumin denaturation inhibition (BSA-DI) from bee pollen collected from various regions of Lithuania. BSA-DI was used as a preliminary in vitro indicator of potential anti-inflammatory activity.

The present study demonstrated that single- and multi-stage bioprocessing can substantially modify the measured bioactive-related properties of bee pollen collected from different regions of Lithuania. In natural bee pollen, TPC, TFC, RSA, and BSA-DI ranged from 5.8 to 13.3 mg/g RUE, 3.7 to 8.4 mg/g RUE, 4.3 to 9.9 mg/g RUE, and 8.7 to 18.8 mg/g SAE, respectively. Among the single-stage treatments, Clara-diastase generally produced the highest values, reaching 16.8–37.85 mg/g RUE for TPC, 11.7–25.9 mg/g RUE for TFC, 10.6–18.6 mg/g RUE for RSA, and 25.0–54.7 mg/g SAE for BSA-DI across the tested pollen samples.

Multi-stage bioprocessing resulted in further increases in the measured parameters. The combination of Clara-diastase and *Lactobacillus acidophilus* produced the highest TPC and BSA-DI values, reaching 28.8–48.6 mg/g RUE and 42.9–70.8 mg/g SAE, respectively, across the tested pollen samples. For TFC and RSA, similarly high values were obtained with Clara-diastase combined with *L. acidophilus* and Viscozyme L combined with *L. acidophilus*, reaching 15.0–35.0 mg/g RUE and 22.1–49.0 mg/g RUE, respectively. The comprehensive statistical analysis proved that observed differences were statistically significant under the tested conditions.

Overall, the findings suggest that combining enzymatic hydrolysis with bacterial fermentation may represent a promising strategy for enhancing the measured bioactive-related properties of bee pollen. However, treatment performance varied depending on the enzyme, microorganism, treatment sequence, and pollen sample. Microbiological safety of the multi-day fermentation processes was not assessed in the present study; therefore, further studies investigating microbial growth, endogenous pollen microbiota, fermentation metabolites, and microbiological safety, together with process mass balance, economic feasibility, and scalability, would be valuable for assessing the safety and practical applicability of the proposed bioprocessing approach.

## Figures and Tables

**Table 1:** Characterization and geographical information of the bee pollen samples collected in Lithuania, including sampling location, sample abbreviation, geographic coordinates (latitude and longitude), and collection period.

No.	Location	Abbreviation	Latitude and Longitude	Collection Period
1.	Klaipėda County, Luoba	KLPD	56°13′ N 21°58′ E	May 2023
2.	Telšiai County, Didieji Mostaičiai	TLS	55°46′ N 21°40′ E	April 2023
3.	Tauragė County	TRG	55°25′ N 22°29′ E	May 2023
4.	Marijampolė County, Matjošiškės	MRMPL	54°59′ N 23°15′ E	June 2023
5.	Šiauliai County, Kurtuvėnai	SL	55°49′ N 23°03′ E	June 2023
6.	Utena County, Antazavė	UTN	55°48′ N 25°55′ E	June 2023
7.	Panevėžys County	PNVZ	55°43′ N 24°21′ E	June 2023
8.	Kaunas County, Paneriai	KNS	54°55′ N 24°45′ E	May 2023
9.	Alytus County, Kalėnai	ALTS	54°23′ N 24°40′ E	June 2023
10.	Vilnius County, Daulėnai	VLNS	54°14′ N 25°44′ E	June 2023

**Table 2:** Bee pollen treatment strategies (enzymatic hydrolysis, fermentation, and combined enzymatic and bacterial treatments) and their durations.

	Treatment Method of Bee Pollen		Abbreviation	Treatment Duration
1	Enzymatic hydrolysis	Cellulase	Cell	3 h 15 min
2		Clara-diastase,	CD	
3		Viscozyme L,	VisL	
4	Fermentation	Spontaneous	Spont	11 days
5		*Lactobacillus acidophilus*	LAcd	7 days
6		*Limosilactobacillus reuteri*	LRtr	8 days
7		*Bifidobacterium animalis*	BAnm	
8		*Lacticaseibacillus rhamnosus*	LRh	9 days
9	Multi-stage bioprocessing	Clara-diastase + *L. rhamnosus*	CD + LRh	4 days
10		Clara-diastase + *B. animalis*	CD + BAnm	
11		Clara-diastase + *L. reuteri*	CD + LRtr	
12		Viscozyme L + *B. animalis*	VisL + BAnm	
13		Viscozyme L + *L. acidophilus*	VisL + LAcd	
14		Viscozyme L + *L. reuteri*	VisL + LRtr	
15		*B. animalis* + Viscozyme L	BAnm + VisL	5 days
16		*B. animalis* + Clara-diastase	BAnm + CD	
17		*L. acidophilus* + Viscozyme L	LAcd + VisL	
18		*L. reuteri* + Clara-diastase	LRtr + CD	
19		*L. reuteri* + Viscozyme L	LRtr + VisL	
20		Viscozyme L + *L. rhamnosus*	VisL + LRh	
21		Viscozyme L + spontaneous fermentation	VisL + Spnt	
22		Clara-diastase + *L. acidophilus*	CD + LAcd	
23		Cellulase + *B. animalis*	Cell + BAnm	
24		Cellulase + *L. reuteri*	Cell + LRtr	
25		Cellulase + *L. rhamnosus*	Cell + LRh	
26		*L. rhamnosus* + Viscozyme L	LRh + VisL	6 days
27		*L. rhamnosus* + Clara-diastase	LRh + CD	
28		*L. acidophilus* + Clara-diastase	LAcd + CD	
29		*L. reuteri* + cellulase	LRtr + Cell	
30		Clara-diastase + spontaneous fermentation	CD + Spnt	
31		Cellulase + spontaneous fermentation	Cell + Spnt	
32		Cellulase + *L. acidophilus*	Cell + LAcd	
33		*B. animalis* + cellulase	BAnm + Cell	7 days
34		*L. acidophilus* + cellulase	LAcd + Cell	
35		*L. rhamnosus* + cellulase	LRh + Cell	
36		Spontaneous fermentation + Clara-diastase	Spnt + CD	
37		Spontaneous fermentation + Viscozyme L	Spnt + VisL	
38		Spontaneous fermentation + cellulase	Spnt + Cell	8 days
39		Natural—no additional extraction, only methanol extraction	Nat	

**Table 3:** Descriptive statistics of bioactive compounds and activities in natural bee pollen samples. Values are expressed as mean ± standard deviation (SD), with minimum (min) and maximum (max) values indicating variability across 10 independent bee pollen samples collected from different regions of Lithuania. Treatment abbreviations are given in Table 2.

Treatment Method	Total Phenolic Content (mg RUE/g)				Total Flavonoid Content (mg RUE/g)				Radical Scavenging Activity (mg RUE/g)				BSA Denaturation Inhibition (mg SAE/g)			
	Mean	SD	Min	Max	Mean	SD	Min	Max	Mean	SD	Min	Max	Mean	SD	Min	Max
Natural	9.2	2.5	5.8	13.3	5.8	1.6	3.7	8.4	6.9	1.9	4.3	9.9	13.1	3.6	8.7	18.8
Spont	11.1	2.5	7.8	14.8	7.8	2.1	5.0	11.2	8.2	2.3	5.2	11.8	16.1	3.7	11.0	22.1
LRh	14.4	4.0	9.3	20.8	9.8	2.7	6.4	14.5	9.0	2.5	6.0	12.9	21.1	5.7	13.7	29.5
LRtr	16.3	4.5	10.7	23.6	9.5	2.6	6.2	13.9	9.6	2.6	6.2	13.7	23.7	6.4	15.6	33.2
BAnm	14.8	4.2	9.4	21.7	9.7	2.7	6.2	14.0	8.8	2.2	6.0	12.4	21.5	5.8	14.1	29.9
LAcd	18.0	5.0	11.5	26.0	10.6	2.9	6.7	15.4	10.3	2.3	6.9	14.0	26.5	7.3	17.1	37.3
Cell	19.4	5.3	12.7	28.2	11.9	3.2	7.8	17.2	11.8	2.6	8.0	16.1	28.6	7.7	18.6	40.0
CD	26.2	7.2	16.8	37.9	18.0	4.9	11.7	25.9	14.0	2.7	10.6	18.6	38.6	10.6	25.1	54.7
VisL	21.5	6.0	14.0	31.2	14.3	3.9	9.3	20.8	12.7	2.5	8.9	16.8	31.7	8.6	20.6	44.6
LRh + Cell	20.9	4.3	14.3	27.7	12.6	3.5	8.0	18.2	13.2	3.4	8.8	18.6	30.8	6.1	21.3	39.3
LRh + CD	30.3	6.1	20.8	40.0	18.6	5.1	11.9	27.0	19.5	5.3	12.4	27.9	44.9	9.0	30.9	57.9
LRh + VisL	22.8	4.6	15.6	30.1	13.8	3.8	9.0	19.9	17.2	4.6	11.0	24.5	33.7	6.7	23.4	43.1
LRtr + Cell	23.5	6.5	15.1	34.0	17.4	4.8	11.2	25.2	23.7	6.4	15.5	33.8	34.6	9.4	22.6	48.9
LRtr + CD	33.8	6.8	23.2	44.5	20.2	5.6	13.0	29.3	27.5	7.4	17.9	39.3	50.3	9.9	34.5	64.8
LRtr + VisL	27.8	7.7	17.9	40.3	18.7	5.2	12.1	27.1	25.3	6.8	16.3	36.1	41.1	11.2	26.9	58.3
BAnm + Cell	20.7	5.7	13.3	30.1	13.0	3.6	8.3	18.9	20.3	5.5	13.2	29.2	30.6	8.3	19.9	43.0
BAnm + CD	30.5	5.4	21.4	38.5	18.0	5.0	11.3	26.0	23.1	6.3	14.9	33.0	45.1	7.7	31.8	55.7
BAnm + VisL	24.9	6.9	16.1	36.1	14.8	4.1	9.7	21.6	21.3	5.8	13.7	30.4	36.7	10.0	23.8	52.0
LAcd + Cell	26.3	5.8	18.0	35.7	18.3	5.1	11.8	26.6	24.6	6.6	15.7	34.9	38.9	8.6	26.2	51.3
LAcd + CD	31.7	6.9	21.5	42.6	23.3	6.3	15.4	33.5	28.4	7.7	18.3	40.5	47.0	10.0	32.0	61.8
LAcd + VisL	29.4	5.9	20.2	38.8	21.2	5.9	13.6	30.8	26.7	7.2	17.4	38.1	43.5	8.5	30.0	55.8
Spnt + Cell	13.5	3.1	9.0	18.5	8.9	2.5	5.6	13.1	9.0	2.4	5.7	12.8	19.5	4.4	13.3	25.9
Spnt + CD	17.4	4.8	11.2	25.1	10.3	2.9	6.6	15.0	11.1	3.1	7.2	15.8	25.8	7.0	16.8	36.1
Spnt + VisL	16.1	4.5	10.4	23.6	9.9	2.7	6.3	14.2	10.3	2.7	6.7	14.6	23.5	6.4	15.3	32.9
CD + Spnt	33.7	6.4	23.3	43.7	17.9	4.9	11.5	25.9	27.3	5.5	19.5	36.1	50.0	9.4	34.7	63.4
CD + LAcd	40.1	6.2	28.8	48.6	24.1	6.7	15.6	35.0	35.2	5.6	26.9	44.4	59.6	8.9	42.9	70.8
CD + BAnm	36.9	5.5	26.7	44.2	20.3	5.6	13.1	29.4	30.7	6.3	21.7	40.9	54.9	7.9	39.8	64.2
CD + LRtr	34.4	6.1	24.1	43.3	19.3	5.3	12.4	27.8	29.6	7.0	19.9	40.9	51.0	8.7	35.8	63.0
CD + LRh	33.0	5.8	23.0	41.6	19.3	5.3	12.4	27.8	28.9	6.8	19.5	39.8	48.8	8.4	34.1	60.2
VisL + Spnt	25.8	6.0	17.2	35.5	16.4	4.5	10.5	23.6	23.6	6.4	15.2	33.7	38.4	8.5	27.6	51.1
VisL + LAcd	34.4	7.2	23.6	45.8	23.1	6.4	15.0	33.7	34.3	9.3	22.1	49.0	51.1	10.4	34.9	66.4
VisL + BAnm	30.5	5.4	21.3	38.5	19.7	5.4	12.7	28.3	25.3	5.4	18.1	34.0	45.0	7.7	31.6	55.3
VisL + LRtr	30.2	7.1	20.2	41.5	18.4	5.0	11.8	26.7	24.1	6.5	15.6	34.4	44.8	10.3	30.1	60.2
VisL + LRh	27.4	5.7	18.6	36.3	16.6	4.6	10.9	24.0	23.8	6.4	15.3	33.7	40.7	8.3	27.8	52.6
Cell + Spnt	21.6	5.9	14.0	31.3	14.0	3.8	9.1	20.2	21.8	5.9	14.4	31.0	32.4	7.9	24.5	44.8
Cell + LAcd	30.3	5.3	21.3	38.1	22.0	5.1	14.7	30.2	29.0	4.6	21.0	35.5	44.9	7.7	31.6	55.3
Cell + BAnm	25.3	4.8	17.5	32.7	17.3	4.8	11.1	25.0	21.7	5.2	14.3	29.6	37.3	6.9	26.3	46.9
Cell + LRtr	27.0	6.1	18.1	36.8	17.5	4.8	11.2	25.3	22.5	6.4	14.3	31.4	39.9	8.8	26.9	52.8
Cell + LRh	24.5	5.0	16.7	32.6	15.4	4.2	9.8	22.4	22.0	6.0	14.2	31.4	36.1	7.2	24.8	46.5

## Data Availability

The original contributions presented in this study are included in the article/Supplementary Materials. Further inquiries can be directed to the corresponding author.

## Supplementary Materials

The following supporting information can be downloaded at https://www.mdpi.com/article/10.3390/foods15173056/s1, Table S1. Changes in total phenolic content of bee pollen samples from different regions of Lithuania after various treatment methods; Table S2. Changes in total flavonoid content of bee pollen samples from different regions of Lithuania after various treatment methods; Table S3. Changes in radical scavenging activity of bee pollen samples from different regions of Lithuania after various treatment methods; Table S4. Changes in inhibition of heat-induced bovine serum albumin denaturation of bee pollen samples from different regions of Lithuania after various treatment methods; Figure S1. Pearson correlation coefficients and scatterplots of total phenolic content (TPC), total flavonoid content (TFC), radical scavenging activity (RSA), and inhibition of heat-induced bovine serum albumin denaturation (BSA-DI); Figure S2. HCA clustering of bee pollen treatments based on total phenolic content (TPC). Treatment abbreviations are given in Table 2; Figure S3. HCA clustering of bee pollen treatments based on total flavonoid content (TFC). Treatment abbreviations are given in Table 2; Figure S4. HCA clustering of bee pollen treatments based on radical scavenging activity (RSA). Treatment abbreviations are given in Table 2; Figure S5. HCA clustering of bee pollen treatments based on inhibition of heat-induced bovine serum albumin denaturation (BSA-DI). Treatment abbreviations are given in Table 2.

## Abbreviations

The following abbreviations are used in this manuscript:

HCA	Hierarchical cluster analysis
BSA-DI	Bovine serum albumin denaturation inhibition
LAB	Lactic acid bacteria
TFC	Total flavonoid content
TPC	Total phenolic content
RUE	Rutin equivalents
RSA	Radical scavenging activity
RSD	Relative standard deviation
SAE	Salicylic acid equivalents
WS	Weighted sum
