# Supplementary material for: Multi-Stage Bioprocessing of Bee Pollen by LAB Fermentation and Enzymatic Hydrolysis: Enhancing Phenolic and Flavonoid Content and Bioactivity
Source: Foods. 2026 Aug 28;15(17):3056. doi: 10.3390/foods15173056 (PMC13564557; doi:10.3390/foods15173056)
Supplement: Supplementary file 1 [file foods-15-03056-s001.zip › foods-4465645-supplementary.pdf]

Multi-Stage Bioprocessing of Bee Pollen by LAB Fermentation and Enzymatic Hydrolysis: Enhancing Phenolic and Flavonoid Content and Bioactivity

Vilma Kaškonienė [1*], Vaida Damulienė [1] and Paulius Kaškonas [2]

[1] *Instrumental Analysis Open Access Centre, Vytautas Magnus University, Lithuania*

[2] *Institute of Metrology, Electrical and Electronics Engineering faculty, Kaunas University of Technology, Lithuania*

* Correspondence: vilma.kaskoniene@vdu.lt (V.K.)

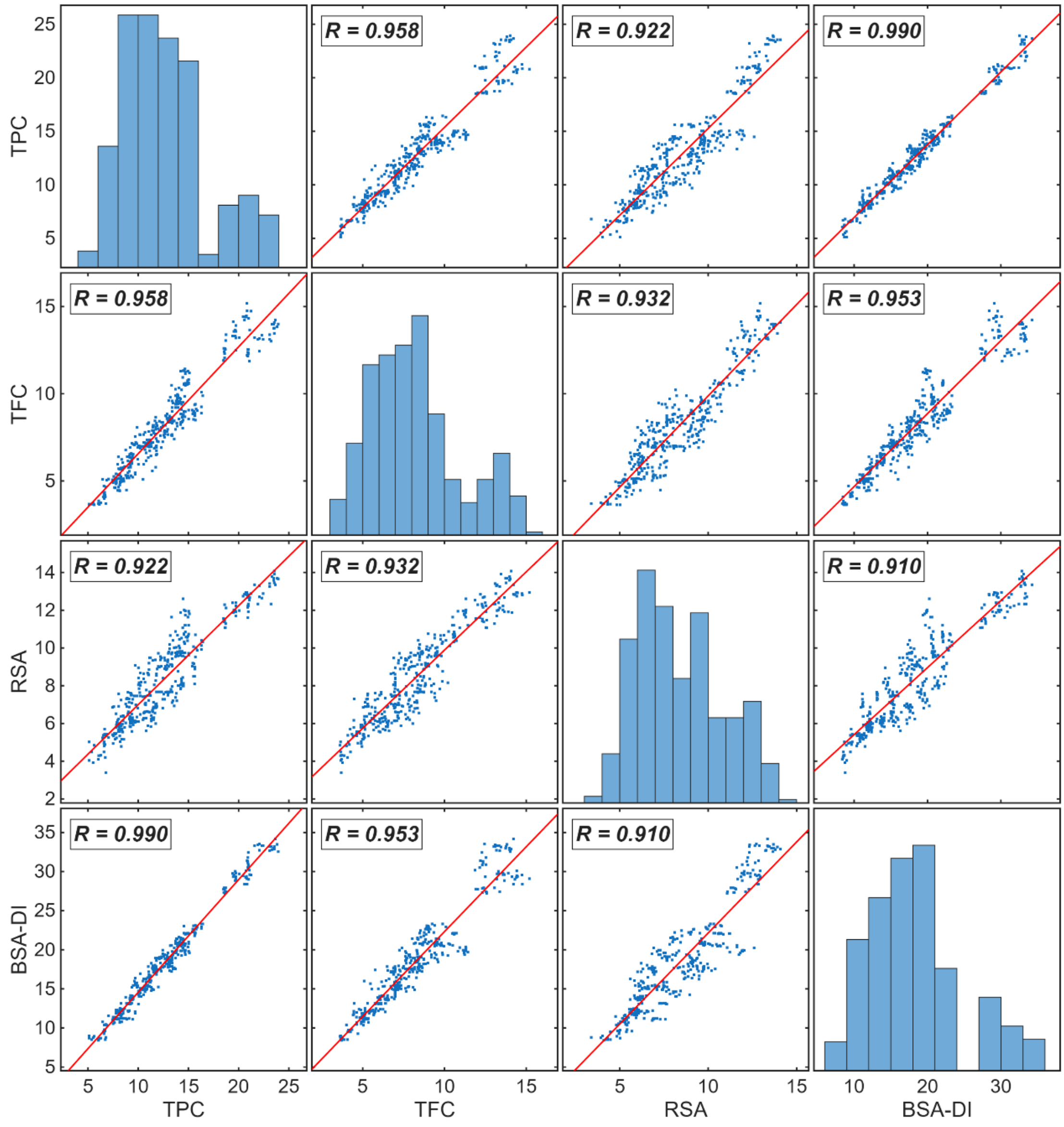

Figure S1. Pearson correlation coefficients and scatterplots of total phenolic content (TPC), total flavonoid content (TFC), radical scavenging activity (RSA), and inhibition of heat-induced bovine serum albumin denaturation (BSA-DI).

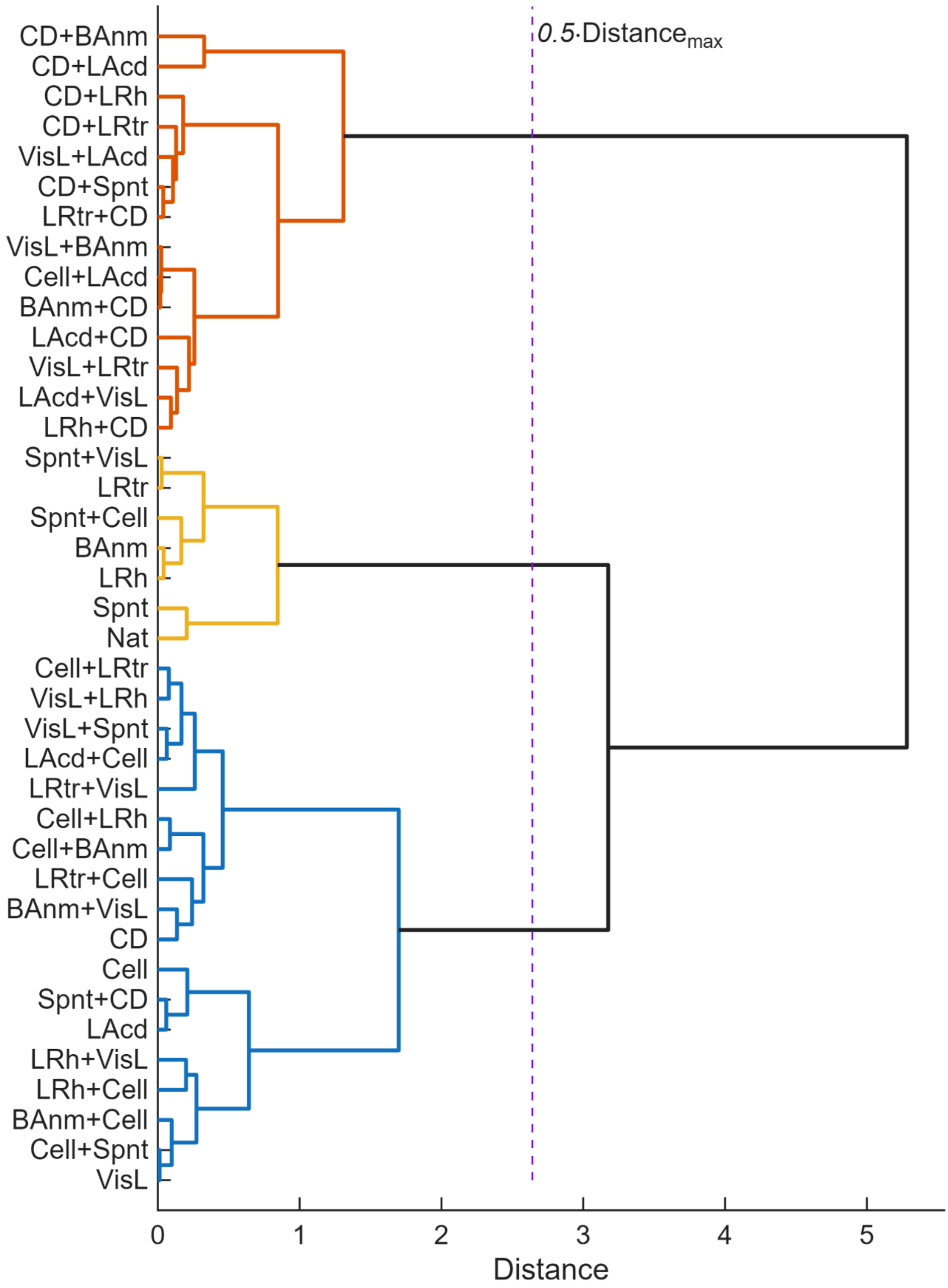

Figure S2. HCA clustering of bee pollen treatments based on total phenolic content (TPC). Treatment abbreviations are given in Table 2.

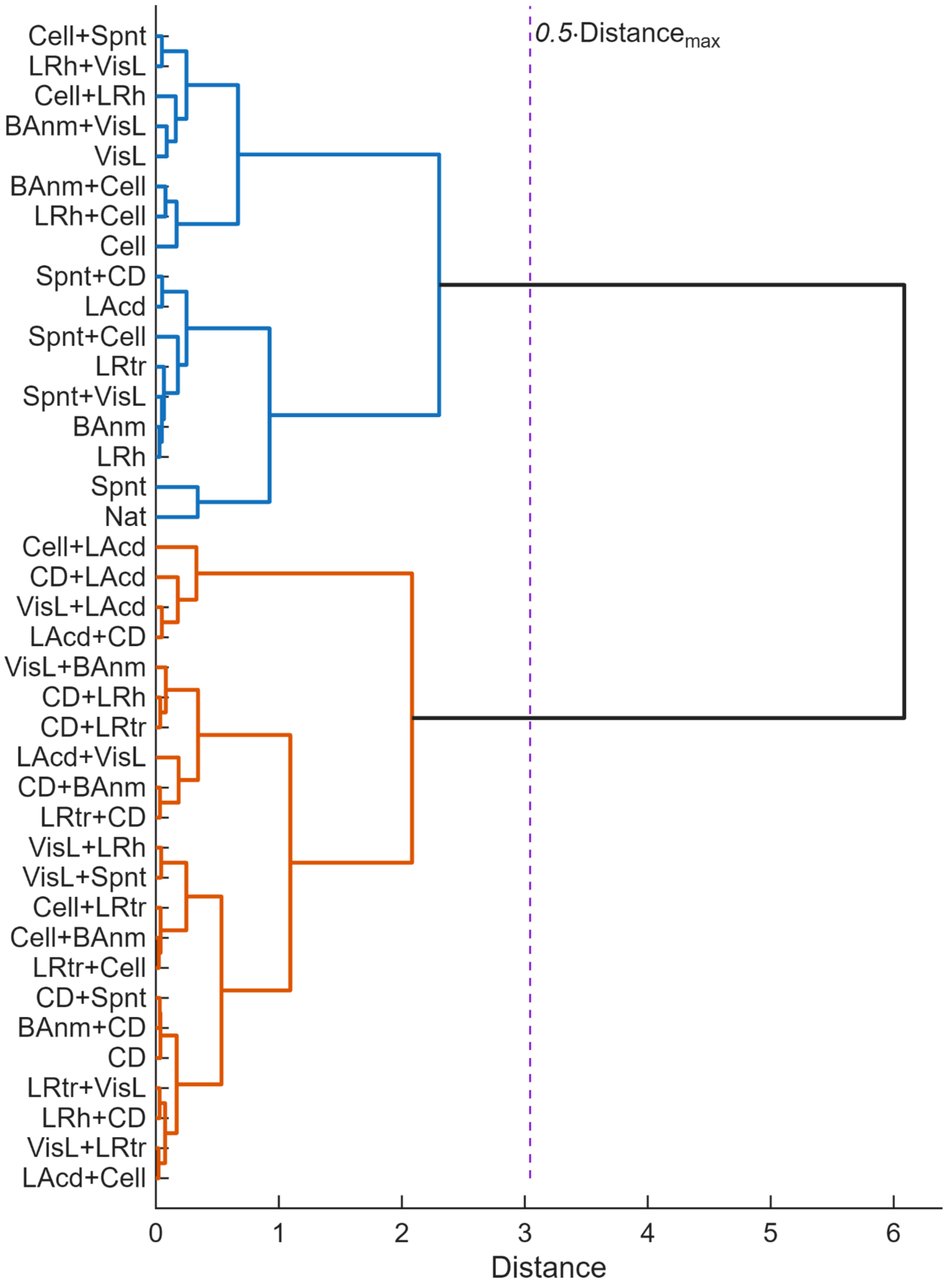

Figure S3. HCA clustering of bee pollen treatments based on total flavonoid content (TFC). Treatment abbreviations are given in Table 2.

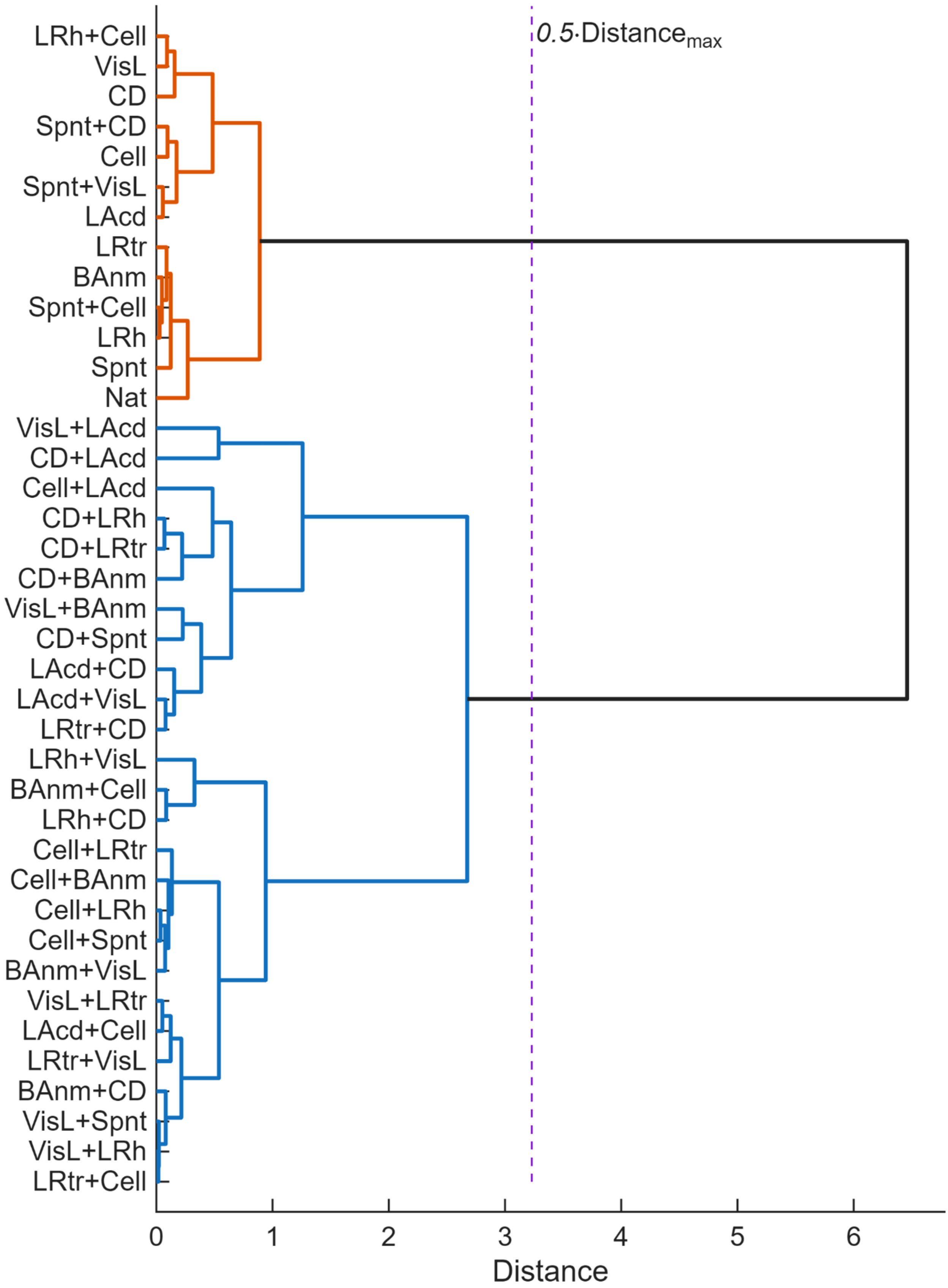

Figure S4. HCA clustering of bee pollen treatments based on radical scavenging activity (RSA). Treatment abbreviations are given in Table 2.

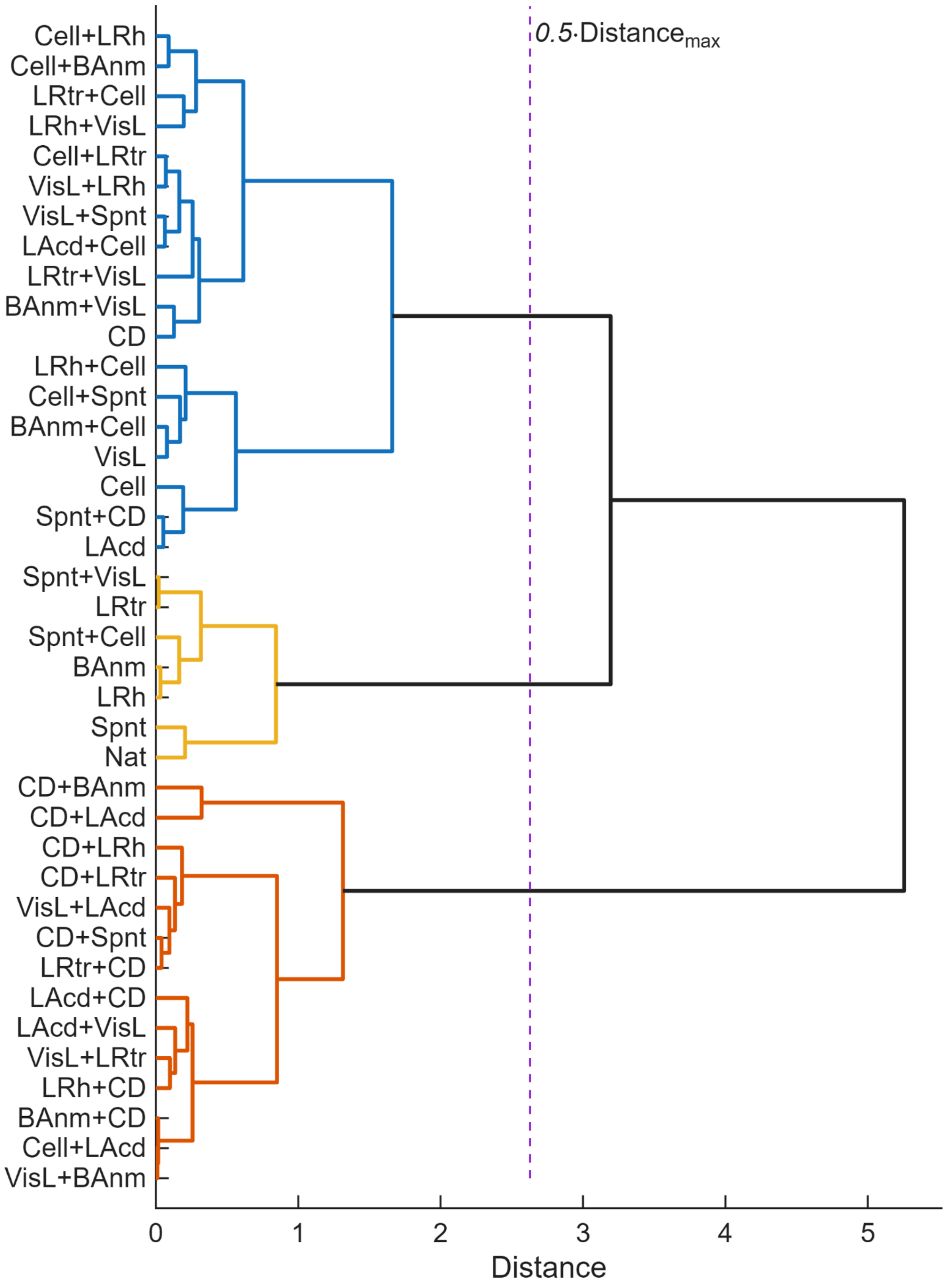

Figure S5. HCA clustering of bee pollen treatments based on inhibition of heat-induced bovine serum albumin denaturation (BSA-DI). Treatment abbreviations are given in Table 2.

Table S1. Changes in total phenolic content of bee pollen samples from different regions of Lithuania after various treatment methods.

Treatment	Total phenolic compounds content of bee pollen samples, mg RUE/g									
	Klaipėda county	Telšiai county	Tauragė county	Marijampolė county	Šiauliai county	Utena county	Panevėžys county	Kaunas county	Alytus county	Vilnius county
**Natural**	7.59 ± 0.65	5.81 ± 0.55	8.76 ± 0.65	8.12 ± 0.17	6.63 ± 0.06	8.41 ± 0.65	9.03 ± 0.32	13.28 ± 0.26	11.89 ± 0.26	12.50 ± 0.46
**Spontaneous fermentation**	9.31 ± 0.09 ^{i}	7.76 ± 0.45 ^{i}	10.89 ± 0.65 ^{i}	10.64 ± 0.54 ^{i}	8.19 ± 0.28 ^{i}	10.25 ± 0.24 ^{i}	10.80 ± 0.49 ^{i}	14.69 ± 0.27 ^{i}	13.90 ± 0.43 ^{i}	14.81 ± 0.18 ^{i}
**Fermentation with *L. rhamnosus***	11.55 ± 0.14 ^{i}	9.30 ± 0.32 ^{i}	13.87 ± 0.35 ^{i}	12.63 ± 0.55 ^{i}	10.31 ± 0.65 ^{i}	13.08 ± 0.25 ^{i}	14.03 ± 0.22 ^{i}	20.83 ± 0.24 ^{i}	18.62 ± 0.08 ^{i}	19.68 ± 0.18 ^{i}
**Fermentation with *L. reuteri***	13.02 ± 0.51 ^{i}	10.71 ± 0.54 ^{i}	15.62 ± 0.19 ^{i}	14.36 ± 0.13 ^{i}	11.79 ± 0.04 ^{i}	14.53 ± 0.35 ^{i}	15.79 ± 0.59 ^{i}	23.62 ± 0.18 ^{i}	20.94 ± 0.06 ^{i}	22.40 ± 0.56 ^{i}
**Fermentation with *B. animalis***	11.85 ± 0.05 ^{i}	9.43 ± 0.10 ^{i}	14.07 ± 0.36 ^{i}	13.04 ± 0.37 ^{i}	10.57 ± 0.22 ^{i}	13.25 ± 0.36 ^{i}	14.34 ± 0.10 ^{i}	21.65 ± 0.32 ^{i}	19.20 ± 0.41 ^{i}	20.21 ± 0.16 ^{i}
**Fermentation with *L. acidophilus***	14.56 ± 0.37 ^{i}	11.50 ± 0.23 ^{i}	17.36 ± 0.14 ^{i}	15.92 ± 0.09 ^{i}	12.95 ± 0.44 ^{i}	16.15 ± 0.46 ^{i}	17.39 ± 0.43 ^{i}	26.00 ± 0.46 ^{i}	23.20 ± 0.07 ^{i}	24.51 ± 0.42 ^{i}
**Enzymatic hydrolysis with cellulase**	15.63 ± 0.23 ^{i}	12.71 ± 0.64 ^{i}	18.42 ± 0.42 ^{i}	17.31 ± 0.65 ^{i}	14.23 ± 0.36 ^{i}	17.57 ± 0.65 ^{i}	18.92 ± 0.35 ^{i}	28.20 ± 0.12 ^{i}	24.75 ± 0.31 ^{i}	26.53 ± 0.59 ^{i}
**Enzymatic hydrolysis with *Clara-diastase***	21.01 ± 0.51 ^{i}	16.84 ± 0.04 ^{i}	25.18 ± 0.30 ^{i}	23.38 ± 0.55 ^{i}	19.20 ± 0.19 ^{i}	23.57 ± 0.45 ^{i}	25.47 ± 0.16 ^{i}	37.85 ± 0.27 ^{i}	33.65 ± 0.06 ^{i}	35.74 ± 0.06 ^{i}
**Enzymatic hydrolysis with *Viscozyme® L***	17.29 ± 0.35 ^{i}	14.04 ± 0.57 ^{i}	20.74 ± 0.17 ^{i}	19.05 ± 0.65 ^{i}	15.33 ± 0.45 ^{i}	19.27 ± 0.50 ^{i}	21.03 ± 0.32 ^{i}	31.23 ± 0.18 ^{i}	27.77 ± 0.40 ^{i}	29.58 ± 0.24 ^{i}
L. rhamnosus + cellulase	17.88 ± 0.13 ^{i}	14.30 ± 0.06 ^{i}	21.44 ± 0.03 ^{i}	19.55 ± 0.36 ^{i}	15.98 ± 0.33 ^{i}	19.85 ± 0.45 ^{i}	21.64 ± 0.05 ^{i}	27.65 ± 0.47 ^{i}	24.34 ± 0.55 ^{i}	26.07 ± 0.19 ^{i}
L. rhamnosus + Clara-diastase	25.92 ± 0.65 ^{i}	20.75 ± 0.23 ^{i}	31.03 ± 0.12 ^{i}	28.34 ± 0.65 ^{i}	23.52 ± 0.44 ^{i}	29.03 ± 0.17 ^{i}	31.36 ± 0.08 ^{i}	39.99 ± 0.18 ^{i}	35.52 ± 0.12 ^{i}	37.72 ± 0.10 ^{i}
L. rhamnosus + Viscozyme® L	19.57 ± 0.13 ^{i}	15.57 ± 0.65 ^{i}	23.41 ± 0.21 ^{i}	21.49 ± 0.18 ^{i}	17.52 ± 0.16 ^{i}	21.94 ± 0.44 ^{i}	23.55 ± 0.28 ^{i}	30.11 ± 0.20 ^{i}	26.58 ± 0.45 ^{i}	28.46 ± 0.65 ^{i}
L. reuteri + cellulase	19.16 ± 0.28 ^{i}	15.07 ± 0.11 ^{i}	22.61 ± 0.12 ^{i}	20.90 ± 0.14 ^{i}	17.02 ± 0.17 ^{i}	21.02 ± 0.45 ^{i}	22.87 ± 0.03 ^{i}	34.00 ± 0.27 ^{i}	30.25 ± 0.18 ^{i}	32.18 ± 0.24 ^{i}
L. reuteri + Clara-diastase	29.03 ± 0.35 ^{i}	23.22 ± 0.19 ^{i}	34.81 ± 0.48 ^{i}	32.00 ± 0.37 ^{i}	25.87 ± 0.65 ^{i}	32.71 ± 0.55 ^{i}	35.28 ± 0.49 ^{i}	44.45 ± 0.21 ^{i}	39.09 ± 0.56 ^{i}	41.86 ± 0.31 ^{i}
L. reuteri + Viscozyme® L	22.41 ± 0.18 ^{i}	17.91 ± 0.35 ^{i}	26.79 ± 0.13 ^{i}	24.50 ± 0.65 ^{i}	20.15 ± 0.06 ^{i}	25.05 ± 0.65 ^{i}	26.96 ± 0.20 ^{i}	40.29 ± 0.22 ^{i}	35.78 ± 0.23 ^{i}	38.06 ± 0.48 ^{i}

B. animalis + cellulase	16.69 ± 0.01 i	13.33 ± 0.02 i	20.02 ± 0.08 i	18.32 ± 0.46 i	14.99 ± 0.36 i	18.69 ± 0.06 i	20.23 ± 0.27 i	30.06 ± 0.04 i	26.73 ± 0.05 i	28.39 ± 0.14 i
B. animalis + Clara-diastase	26.55 ± 0.34 i	21.36 ± 0.56 i	32.09 ± 0.54 i	29.57 ± 0.59 i	24.04 ± 0.37 i	29.78 ± 0.23 i	32.37 ± 0.35 i	38.48 ± 0.63 i	34.15 ± 0.23 i	36.31 ± 0.62 i
B. animalis + Viscozyme® L	19.98 ± 0.22 i	16.14 ± 0.27 i	24.01 ± 0.22 i	22.02 ± 0.01 i	18.02 ± 0.01 i	22.26 ± 0.61 i	24.15 ± 0.44 i	36.09 ± 0.41 i	32.04 ± 0.03 i	34.06 ± 0.43 i
L. acidophilus + cellulase	21.97 ± 0.45 i	18.02 ± 0.65 i	26.37 ± 0.59 i	24.43 ± 0.42 i	19.89 ± 0.16 i	24.76 ± 0.23 i	26.76 ± 0.25 i	35.73 ± 0.60 i	31.50 ± 0.49 i	33.79 ± 0.17 i
L. acidophilus + Clara-diastase	26.77 ± 0.13 i	21.49 ± 0.10 i	32.18 ± 0.08 i	29.45 ± 0.27 i	24.16 ± 0.40 i	30.13 ± 0.28 i	32.47 ± 0.05 i	42.62 ± 0.12 i	37.82 ± 0.13 i	40.29 ± 0.53 i
L. acidophilus + Viscozyme® L	25.30 ± 0.19 i	20.16 ± 0.29 i	30.23 ± 0.18 i	27.73 ± 0.10 i	22.69 ± 0.22 i	28.27 ± 0.19 i	30.70 ± 0.40 i	38.78 ± 0.37 i	34.32 ± 0.20 i	36.22 ± 0.65 i
**Spontaneous +** *cellulase*	11.16 ± 0.65 i	9.01 ± 0.06 i	13.60 ± 0.47 i	12.40 ± 0.39 i	10.14 ± 0.10 i	12.57 ± 0.15 i	13.67 ± 0.35 i	18.45 ± 0.43 i	16.31 ± 0.27 i	17.38 ± 0.42 i
**Spontaneous +** *Clara-diastase*	13.79 ± 0.31 i	11.19 ± 0.29 i	16.73 ± 0.46 i	15.48 ± 0.15 i	12.59 ± 0.21 i	16.11 ± 0.47 i	17.12 ± 0.16 i	25.10 ± 0.20 i	22.23 ± 0.50 i	23.96 ± 0.14 i
**Spontaneous +** *Viscozyme® L*	12.86 ± 0.65 i	10.36 ± 0.34 i	15.61 ± 0.17 i	14.28 ± 0.29 i	11.61 ± 0.50 i	14.64 ± 0.35 i	15.52 ± 0.27 i	23.62 ± 0.29 i	20.62 ± 0.19 i	21.92 ± 0.62 i
Clara-diastase + **Spontaneous**	29.32 ± 0.44 i	23.32 ± 0.27 i	34.84 ± 0.42 i	32.03 ± 0.29 i	26.23 ± 0.09 i	32.42 ± 0.13 i	34.95 ± 0.37 i	43.69 ± 0.49 i	38.79 ± 0.23 i	41.18 ± 0.41 i
Clara-diastase + L. acidophilus	35.89 ± 0.23 i	28.80 ± 0.06 i	43.18 ± 0.04 i	39.49 ± 0.53 i	32.16 ± 0.65 i	40.26 ± 0.26 i	43.54 ± 0.59 i	48.58 ± 0.46 i	43.38 ± 0.53 i	45.86 ± 0.02 i
Clara-diastase + B. animalis	33.24 ± 0.38 i	26.65 ± 0.23 i	39.90 ± 0.19 i	36.66 ± 0.05 i	29.99 ± 0.06 i	37.32 ± 0.03 i	40.39 ± 0.13 i	44.15 ± 0.22 i	39.25 ± 0.20 i	41.76 ± 0.46 i
Clara-diastase + L. reuteri	30.17 ± 0.42 i	24.11 ± 0.04 i	36.17 ± 0.65 i	33.19 ± 0.54 i	27.03 ± 0.28 i	33.70 ± 0.42 i	36.50 ± 0.27 i	43.30 ± 0.50 i	38.66 ± 0.38 i	41.30 ± 0.65 i
Clara-diastase + L. rhamnosus	28.99 ± 0.37 i	23.03 ± 0.10 i	34.80 ± 0.35 i	31.76 ± 0.04 i	25.93 ± 0.14 i	32.41 ± 0.16 i	34.93 ± 0.15 i	41.63 ± 0.40 i	36.95 ± 0.04 i	39.22 ± 0.18 i
Viscozyme® L + **Spontaneous**	21.68 ± 0.39 i	17.18 ± 0.06 i	25.81 ± 0.16 i	23.57 ± 0.65 i	19.35 ± 0.02 i	24.03 ± 0.62 i	26.03 ± 0.17 i	35.46 ± 0.10 i	31.54 ± 0.27 i	33.59 ± 0.20 i
Viscozyme® L + L. acidophilus	29.37 ± 0.18 i	23.57 ± 0.49 i	35.03 ± 0.22 i	32.04 ± 0.25 i	26.45 ± 0.65 i	32.79 ± 0.63 i	35.47 ± 0.05 i	45.77 ± 0.11 i	40.68 ± 0.37 i	43.20 ± 0.09 i

Viscozyme® L + B. animalis	26.66 ± 0.10 i	21.33 ± 0.59 i	32.40 ± 0.42 i	29.35 ± 0.03 i	23.87 ± 0.43 i	29.89 ± 0.12 i	32.49 ± 0.42 i	38.48 ± 0.30 i	33.82 ± 0.44 i	36.55 ± 0.37 i
Viscozyme® L + L. reuteri	25.10 ± 0.39 i	20.18 ± 0.07 i	30.31 ± 0.60 i	27.77 ± 0.22 i	22.66 ± 0.13 i	28.22 ± 0.03 i	30.50 ± 0.49 i	41.54 ± 0.39 i	36.85 ± 0.19 i	39.31 ± 0.18 i
Viscozyme® L + L. rhamnosus	23.39 ± 0.25 i	18.58 ± 0.44 i	28.05 ± 0.25 i	25.77 ± 0.04 i	21.18 ± 0.22 i	26.28 ± 0.52 i	28.10 ± 0.38 i	36.31 ± 0.24 i	32.43 ± 0.38 i	34.32 ± 0.28 i
***Cellulase* + Spontaneous**	17.32 ± 0.09 i	14.03 ± 0.25 i	20.94 ± 0.16 i	19.00 ± 0.21 i	15.56 ± 0.22 i	19.29 ± 0.43 i	21.23 ± 0.41 i	31.27 ± 0.47 i	27.75 ± 0.39 i	29.46 ± 0.26 i
Cellulase + L. acidophilus	26.59 ± 0.08 i	21.28 ± 0.57 i	31.89 ± 0.11 i	29.34 ± 0.65 i	23.91 ± 0.04 i	29.79 ± 0.19 i	32.13 ± 0.48 i	38.11 ± 0.52 i	34.08 ± 0.21 i	36.20 ± 0.62 i
Cellulase + B. animalis	21.80 ± 0.49 i	17.53 ± 0.38 i	26.01 ± 0.25 i	24.00 ± 0.03 i	19.64 ± 0.02 i	24.35 ± 0.57 i	26.36 ± 0.64 i	32.72 ± 0.15 i	29.18 ± 0.38 i	30.98 ± 0.39 i
Cellulase + L. reuteri	22.57 ± 0.09 i	18.13 ± 0.38 i	26.79 ± 0.42 i	24.93 ± 0.29 i	20.34 ± 0.44 i	25.35 ± 0.22 i	27.37 ± 0.02 i	36.76 ± 0.40 i	32.59 ± 0.13 i	34.75 ± 0.37 i
Cellulase + L. rhamnosus	20.88 ± 0.15 i	16.73 ± 0.19 i	25.04 ± 0.27 i	22.99 ± 0.10 i	18.93 ± 0.41 i	23.33 ± 0.31 i	25.27 ± 0.27 i	32.56 ± 0.43 i	28.61 ± 0.54 i	30.64 ± 0.21 i

Note. Values are expressed as rutin equivalents (mg RUE/g bee pollen) and represent the mean of 10 technical replicate measurements per sample. “i” denotes a statistically significant increase ($p < 0.05$).

Table S2. Changes in total flavonoid content of bee pollen samples from different regions of Lithuania after various treatment methods.

Treatment	Total flavonoids content of bee pollen samples, mg RUE/g									
	Klaipėda county	Telšiai county	Tauragė county	Marijampolė county	Šiauliai county	Utena county	Panevėžys county	Kaunas county	Alytus county	Vilnius county
**Natural**	4.87 ± 0.29	3.70 ± 0.07	5.59 ± 0.15	4.96 ± 0.37	4.21 ± 0.20	5.22 ± 0.10	5.72 ± 0.56	8.44 ± 0.19	7.48 ± 0.65	7.82 ± 0.21
**Spontaneous fermentation**	6.32 ± 0.30 ^{i}	4.99 ± 0.07 ^{i}	7.39 ± 0.51 ^{i}	7.09 ± 0.51 ^{i}	5.60 ± 0.58 ^{i}	6.99 ± 0.01 ^{i}	7.54 ± 0.50 ^{i}	11.20 ± 0.19 ^{i}	9.90 ± 0.36 ^{i}	10.59 ± 0.07 ^{i}
**Fermentation with *L. rhamnosus***	7.98 ± 0.23 ^{i}	6.35 ± 0.28 ^{i}	9.48 ± 0.48 ^{i}	8.52 ± 0.31 ^{i}	7.11 ± 0.09 ^{i}	8.65 ± 0.65 ^{i}	9.51 ± 0.16 ^{i}	14.46 ± 0.40 ^{i}	12.52 ± 0.44 ^{i}	13.44 ± 0.45 ^{i}
**Fermentation with *L. reuteri***	7.87 ± 0.41 ^{i}	6.18 ± 0.65 ^{i}	8.85 ± 0.25 ^{i}	8.58 ± 0.29 ^{i}	6.78 ± 0.44 ^{i}	8.70 ± 0.40 ^{i}	9.23 ± 0.44 ^{i}	13.90 ± 0.21 ^{i}	12.27 ± 0.22 ^{i}	13.09 ± 0.26 ^{i}
**Fermentation with *B. animalis***	7.80 ± 0.04 ^{i}	6.20 ± 0.14 ^{i}	9.36 ± 0.18 ^{i}	8.60 ± 0.08 ^{i}	7.03 ± 0.12 ^{i}	8.67 ± 0.21 ^{i}	9.28 ± 0.37 ^{i}	14.02 ± 0.33 ^{i}	12.57 ± 0.19 ^{i}	13.34 ± 0.65 ^{i}
**Fermentation with *L. acidophilus***	8.56 ± 0.02 ^{i}	6.71 ± 0.11 ^{i}	10.33 ± 0.17 ^{i}	9.36 ± 0.14 ^{i}	7.64 ± 0.65 ^{i}	9.50 ± 0.16 ^{i}	10.20 ± 0.22 ^{i}	15.36 ± 0.25 ^{i}	13.60 ± 0.23 ^{i}	14.46 ± 0.36 ^{i}
**Enzymatic hydrolysis with cellulase**	9.65 ± 0.28 ^{i}	7.82 ± 0.52 ^{i}	11.60 ± 0.52 ^{i}	10.52 ± 0.06 ^{i}	8.61 ± 0.04 ^{i}	10.72 ± 0.03 ^{i}	11.69 ± 0.20 ^{i}	17.15 ± 0.54 ^{i}	15.08 ± 0.55 ^{i}	16.26 ± 0.09 ^{i}
**Enzymatic hydrolysis with *Clara-diastase***	14.55 ± 0.32 ^{i}	11.65 ± 0.11 ^{i}	17.40 ± 0.14 ^{i}	16.03 ± 0.25 ^{i}	13.10 ± 0.08 ^{i}	16.48 ± 0.35 ^{i}	17.65 ± 0.31 ^{i}	25.92 ± 0.38 ^{i}	23.12 ± 0.35 ^{i}	24.56 ± 0.53 ^{i}
**Enzymatic hydrolysis with *Viscozyme® L***	11.57 ± 0.11 ^{i}	9.28 ± 0.15 ^{i}	14.02 ± 0.55 ^{i}	12.72 ± 0.25 ^{i}	10.16 ± 0.44 ^{i}	13.03 ± 0.46 ^{i}	14.00 ± 0.65 ^{i}	20.75 ± 0.16 ^{i}	18.20 ± 0.62 ^{i}	19.61 ± 0.14 ^{i}
L. rhamnosus + cellulase	10.05 ± 0.15 ^{i}	8.04 ± 0.05 ^{i}	12.13 ± 0.05 ^{i}	11.22 ± 0.36 ^{i}	9.06 ± 0.18 ^{i}	11.12 ± 0.46 ^{i}	12.28 ± 0.40 ^{i}	18.22 ± 0.56 ^{i}	15.97 ± 0.30 ^{i}	17.46 ± 0.51 ^{i}
L. rhamnosus + Clara-diastase	14.92 ± 0.30 ^{i}	11.92 ± 0.55 ^{i}	17.99 ± 0.32 ^{i}	16.40 ± 0.27 ^{i}	13.69 ± 0.65 ^{i}	16.71 ± 0.14 ^{i}	18.13 ± 0.08 ^{i}	26.99 ± 0.26 ^{i}	23.90 ± 0.22 ^{i}	25.43 ± 0.13 ^{i}
L. rhamnosus + Viscozyme® L	11.14 ± 0.60 ^{i}	9.03 ± 0.21 ^{i}	13.33 ± 0.10 ^{i}	12.37 ± 0.36 ^{i}	10.01 ± 0.51 ^{i}	12.29 ± 0.44 ^{i}	13.35 ± 0.49 ^{i}	19.93 ± 0.47 ^{i}	17.84 ± 0.31 ^{i}	18.87 ± 0.09 ^{i}
L. reuteri + cellulase	13.94 ± 0.13 ^{i}	11.17 ± 0.06 ^{i}	16.71 ± 0.52 ^{i}	15.51 ± 0.40 ^{i}	12.48 ± 0.49 ^{i}	15.67 ± 0.08 ^{i}	16.88 ± 0.48 ^{i}	25.20 ± 0.16 ^{i}	22.35 ± 0.10 ^{i}	23.88 ± 0.55 ^{i}
L. reuteri + Clara-diastase	16.04 ± 0.52 ^{i}	12.95 ± 0.43 ^{i}	19.81 ± 0.65 ^{i}	17.87 ± 0.22 ^{i}	14.69 ± 0.31 ^{i}	18.35 ± 0.43 ^{i}	19.75 ± 0.19 ^{i}	29.33 ± 0.12 ^{i}	25.86 ± 0.65 ^{i}	27.76 ± 0.32 ^{i}
L. reuteri + Viscozyme® L	14.96 ± 0.65 ^{i}	12.13 ± 0.17 ^{i}	18.07 ± 0.13 ^{i}	16.48 ± 0.04 ^{i}	13.54 ± 0.65 ^{i}	16.83 ± 0.19 ^{i}	18.03 ± 0.40 ^{i}	27.06 ± 0.29 ^{i}	24.06 ± 0.08 ^{i}	25.88 ± 0.65 ^{i}
B. animalis + cellulase	10.34 ± 0.56 ^{i}	8.26 ± 0.45 ^{i}	12.63 ± 0.57 ^{i}	11.50 ± 0.05 ^{i}	9.40 ± 0.08 ^{i}	11.63 ± 0.16 ^{i}	12.91 ± 0.39 ^{i}	18.92 ± 0.49 ^{i}	16.57 ± 0.30 ^{i}	17.74 ± 0.14 ^{i}

B. animalis* + *Clara-diastase	14.55 ± 0.62 i	11.34 ± 0.46 i	17.34 ± 0.35 i	15.89 ± 0.56 i	13.10 ± 0.07 i	16.15 ± 0.43 i	17.79 ± 0.50 i	26.00 ± 0.20 i	23.09 ± 0.49 i	24.67 ± 0.24 i
B. animalis* + *Viscozyme® L	11.89 ± 0.20 i	9.67 ± 0.53 i	14.40 ± 0.65 i	12.90 ± 0.47 i	10.66 ± 0.09 i	13.39 ± 0.08 i	14.39 ± 0.28 i	21.64 ± 0.52 i	19.12 ± 0.42 i	20.25 ± 0.35 i
L. acidophilus* + *cellulase	14.88 ± 0.09 i	11.79 ± 0.21 i	17.76 ± 0.06 i	16.16 ± 0.36 i	13.27 ± 0.43 i	16.46 ± 0.21 i	17.86 ± 0.17 i	26.55 ± 0.17 i	23.59 ± 0.60 i	25.16 ± 0.13 i
L. acidophilus* + *Clara-diastase	18.81 ± 0.58 i	15.35 ± 0.65 i	22.60 ± 0.31 i	20.55 ± 0.25 i	16.88 ± 0.22 i	20.98 ± 0.48 i	22.68 ± 0.11 i	33.51 ± 0.56 i	29.98 ± 0.65 i	31.88 ± 0.24 i
L. acidophilus* + *Viscozyme® L	16.99 ± 0.49 i	13.62 ± 0.33 i	20.52 ± 0.10 i	18.77 ± 0.25 i	15.35 ± 0.24 i	19.17 ± 0.43 i	20.30 ± 0.63 i	30.79 ± 0.30 i	27.31 ± 0.07 i	29.04 ± 0.06 i
**Spontaneous + *cellulase***	7.31 ± 0.49 i	5.61 ± 0.41 i	8.37 ± 0.57 i	7.90 ± 0.25 i	6.56 ± 0.44 i	8.13 ± 0.15 i	8.65 ± 0.37 i	13.14 ± 0.30 i	11.47 ± 0.40 i	12.23 ± 0.59 i
**Spontaneous + *Clara-diastase***	8.31 ± 0.26 i	6.58 ± 0.19 i	9.99 ± 0.05 i	9.09 ± 0.23 i	7.25 ± 0.65 i	9.25 ± 0.17 i	10.13 ± 0.45 i	15.01 ± 0.34 i	13.37 ± 0.34 i	14.13 ± 0.03 i
**Spontaneous + *Viscozyme® L***	8.14 ± 0.32 i	6.34 ± 0.11 i	9.59 ± 0.08 i	8.87 ± 0.43 i	7.16 ± 0.25 i	9.02 ± 0.32 i	9.63 ± 0.13 i	14.17 ± 0.55 i	12.92 ± 0.63 i	13.62 ± 0.09 i
***Clara-diastase* + Spontaneous**	14.42 ± 0.18 i	11.54 ± 0.12 i	17.39 ± 0.64 i	15.75 ± 0.55 i	13.01 ± 0.36 i	16.06 ± 0.56 i	17.41 ± 0.28 i	25.94 ± 0.49 i	23.01 ± 0.19 i	24.50 ± 0.14 i
Clara-diastase* + *L. acidophilus	19.37 ± 0.34 i	15.56 ± 0.39 i	23.38 ± 0.18 i	21.07 ± 0.62 i	17.47 ± 0.03 i	21.79 ± 0.28 i	23.57 ± 0.52 i	34.96 ± 0.17 i	31.11 ± 0.51 i	33.00 ± 0.23 i
Clara-diastase* + *B. animalis	16.31 ± 0.36 i	13.14 ± 0.15 i	19.57 ± 0.09 i	17.91 ± 0.27 i	14.53 ± 0.46 i	18.36 ± 0.60 i	19.72 ± 0.35 i	29.43 ± 0.18 i	26.18 ± 0.29 i	27.70 ± 0.16 i
Clara-diastase* + *L. reuteri	15.51 ± 0.17 i	12.44 ± 0.14 i	18.85 ± 0.23 i	17.13 ± 0.13 i	14.02 ± 0.03 i	17.36 ± 0.43 i	18.86 ± 0.49 i	27.78 ± 0.33 i	24.86 ± 0.08 i	26.40 ± 0.29 i
Clara-diastase* + *L. rhamnosus	15.61 ± 0.20 i	12.40 ± 0.46 i	18.47 ± 0.27 i	17.04 ± 0.10 i	13.82 ± 0.49 i	17.68 ± 0.65 i	18.71 ± 0.11 i	27.82 ± 0.37 i	24.71 ± 0.42 i	26.28 ± 0.17 i
***Viscozyme® L* + Spontaneous**	13.14 ± 0.03 i	10.52 ± 0.08 i	15.63 ± 0.60 i	14.48 ± 0.05 i	11.77 ± 0.56 i	15.00 ± 0.42 i	15.89 ± 0.03 i	23.57 ± 0.32 i	21.16 ± 0.37 i	22.51 ± 0.35 i
Viscozyme® L* + *L. acidophilus	18.58 ± 0.06 i	14.95 ± 0.34 i	22.32 ± 0.18 i	20.44 ± 0.28 i	16.72 ± 0.10 i	20.86 ± 0.09 i	22.47 ± 0.43 i	33.66 ± 0.38 i	29.66 ± 0.35 i	31.56 ± 0.65 i
Viscozyme® L* + *B. animalis	15.78 ± 0.11 i	12.65 ± 0.13 i	19.12 ± 0.58 i	17.44 ± 0.56 i	14.33 ± 0.38 i	17.48 ± 0.65 i	19.22 ± 0.41 i	28.25 ± 0.44 i	25.40 ± 0.20 i	26.99 ± 0.43 i
Viscozyme® L* + *L. reuteri	14.90 ± 0.44 i	11.80 ± 0.18 i	17.72 ± 0.60 i	16.23 ± 0.05 i	13.30 ± 0.05 i	16.70 ± 0.21 i	17.84 ± 0.16 i	26.66 ± 0.62 i	23.81 ± 0.38 i	24.85 ± 0.45 i

Viscozyme® L + L. rhamnosus	13.20 ± 0.57 i	10.85 ± 0.27 i	15.97 ± 0.31 i	14.68 ± 0.65 i	11.89 ± 0.26 i	14.94 ± 0.18 i	15.84 ± 0.65 i	23.97 ± 0.13 i	21.34 ± 0.05 i	22.86 ± 0.52 i
***Cellulase* + Spontaneous**	11.14 ± 0.65 i	9.11 ± 0.27 i	13.51 ± 0.29 i	12.28 ± 0.48 i	10.49 ± 0.59 i	12.62 ± 0.62 i	13.55 ± 0.33 i	20.23 ± 0.11 i	18.14 ± 0.54 i	19.16 ± 0.25 i
Cellulase + L. acidophilus	18.16 ± 0.49 i	14.66 ± 0.15 i	22.11 ± 0.11 i	20.07 ± 0.39 i	16.55 ± 0.16 i	20.89 ± 0.59 i	22.17 ± 0.08 i	30.23 ± 0.62 i	27.00 ± 0.12 i	28.48 ± 0.18 i
Cellulase + B. animalis	13.91 ± 0.19 i	11.09 ± 0.47 i	16.57 ± 0.44 i	15.36 ± 0.17 i	12.52 ± 0.04 i	15.62 ± 0.56 i	16.68 ± 0.25 i	24.96 ± 0.55 i	22.55 ± 0.24 i	23.66 ± 0.36 i
Cellulase + L. reuteri	14.07 ± 0.07 i	11.22 ± 0.29 i	16.77 ± 0.41 i	15.55 ± 0.48 i	12.73 ± 0.38 i	15.56 ± 0.39 i	17.16 ± 0.33 i	25.33 ± 0.63 i	22.50 ± 0.30 i	23.79 ± 0.16 i
Cellulase + L. rhamnosus	12.36 ± 0.08 i	9.84 ± 0.11 i	14.89 ± 0.65 i	13.59 ± 0.08 i	11.22 ± 0.42 i	14.05 ± 0.34 i	14.94 ± 0.05 i	22.35 ± 0.15 i	19.66 ± 0.43 i	20.86 ± 0.56 i

Note. Values are expressed as rutin equivalents (mg RUE/g bee pollen) and represent the mean of 10 technical replicate measurements per sample. "i" denotes a statistically significant increase ($p < 0.05$).

Table S3. Changes in radical scavenging activity of bee pollen samples from different regions of Lithuania after various treatment methods.

Treatment	Radical scavenging activity of bee pollen samples, mg RUE/g									
	**Klaipėda county**	**Telšiai county**	**Tauragė county**	**Marijampolė county**	**Šiauliai county**	**Utena county**	**Panevėžys county**	**Kaunas county**	**Alytus county**	**Vilnius county**
**Natural**	5.34 ± 0.11	4.27 ± 0.46	6.32 ± 0.34	5.85 ± 0.17	4.98 ± 0.44	6.93 ± 0.36	7.43 ± 0.15	9.86 ± 0.29	8.93 ± 0.55	9.14 ± 0.36
**Spontaneous fermentation**	6.19 ± 0.10 ^{i}	5.24 ± 0.31 ^{i}	7.46 ± 0.18 ^{i}	6.89 ± 0.50 ^{i}	5.79 ± 0.18 ^{i}	8.16 ± 0.40 ^{i}	8.93 ± 0.36 ^{i}	11.79 ± 0.43 ^{i}	10.50 ± 0.52 ^{i}	10.72 ± 0.30 ^{i}
**Fermentation with *L. rhamnosus***	6.52 ± 0.32 ^{i}	6.01 ± 0.25 ^{i}	7.93 ± 0.47 ^{i}	7.66 ± 0.01 ^{i}	6.66 ± 0.40 ^{i}	9.08 ± 0.11 ^{i}	9.70 ± 0.14 ^{i}	12.85 ± 0.09 ^{i}	11.53 ± 0.40 ^{i}	12.04 ± 0.23 ^{i}
**Fermentation with *L. reuteri***	7.28 ± 0.33 ^{i}	6.17 ± 0.37 ^{i}	8.92 ± 0.43 ^{i}	8.10 ± 0.16 ^{i}	6.96 ± 0.12 ^{i}	9.64 ± 0.09 ^{i}	10.21 ± 0.32 ^{i}	13.72 ± 0.23 ^{i}	12.23 ± 0.39 ^{i}	12.89 ± 0.41 ^{i}
**Fermentation with *B. animalis***	7.19 ± 0.47 ^{i}	5.96 ± 0.30 ^{i}	8.18 ± 0.36 ^{i}	7.37 ± 0.05 ^{i}	6.24 ± 0.51 ^{i}	8.57 ± 0.65 ^{i}	9.27 ± 0.65 ^{i}	12.43 ± 0.65 ^{i}	11.07 ± 0.35 ^{i}	11.42 ± 0.44 ^{i}
**Fermentation with *L. acidophilus***	8.26 ± 0.65 ^{i}	6.90 ± 0.62 ^{i}	9.68 ± 0.37 ^{i}	8.97 ± 0.10 ^{i}	7.78 ± 0.23 ^{i}	10.66 ± 0.11 ^{i}	11.59 ± 0.35 ^{i}	14.02 ± 0.65 ^{i}	12.18 ± 0.36 ^{i}	12.63 ± 0.13 ^{i}
**Enzymatic hydrolysis with cellulase**	9.60 ± 0.53 ^{i}	8.04 ± 0.26 ^{i}	11.61 ± 0.09 ^{i}	10.82 ± 0.65 ^{i}	8.93 ± 0.40 ^{i}	11.23 ± 0.37 ^{i}	12.07 ± 0.65 ^{i}	16.10 ± 0.41 ^{i}	14.34 ± 0.32 ^{i}	14.81 ± 0.34 ^{i}
**Enzymatic hydrolysis with *Clara-diastase***	12.34 ± 0.44 ^{i}	10.68 ± 0.13 ^{i}	13.72 ± 0.10 ^{i}	12.79 ± 0.53 ^{i}	10.57 ± 0.52 ^{i}	13.81 ± 0.27 ^{i}	13.77 ± 0.32 ^{i}	18.58 ± 0.49 ^{i}	16.46 ± 0.23 ^{i}	17.13 ± 0.26 ^{i}
**Enzymatic hydrolysis with *Viscozyme® L***	10.58 ± 0.37 ^{i}	8.91 ± 0.29 ^{i}	13.05 ± 0.63 ^{i}	11.78 ± 0.09 ^{i}	9.98 ± 0.48 ^{i}	12.28 ± 0.22 ^{i}	13.46 ± 0.22 ^{i}	16.76 ± 0.20 ^{i}	14.96 ± 0.33 ^{i}	15.67 ± 0.34 ^{i}
L. rhamnosus* + *cellulase	10.28 ± 0.18 ^{i}	8.80 ± 0.37 ^{i}	12.55 ± 0.08 ^{i}	11.70 ± 0.52 ^{i}	9.71 ± 0.46 ^{i}	12.53 ± 0.64 ^{i}	13.30 ± 0.02 ^{i}	18.60 ± 0.65 ^{i}	16.84 ± 0.16 ^{i}	17.53 ± 0.27 ^{i}
L. rhamnosus* + *Clara-diastase	14.75 ± 0.40 ^{i}	12.42 ± 0.42 ^{i}	18.08 ± 0.11 ^{i}	16.62 ± 0.58 ^{i}	14.01 ± 0.29 ^{i}	19.56 ± 0.03 ^{i}	21.18 ± 0.32 ^{i}	27.90 ± 0.45 ^{i}	24.68 ± 0.51 ^{i}	25.93 ± 0.42 ^{i}
L. rhamnosus* + *Viscozyme® L	13.00 ± 0.11 ^{i}	10.98 ± 0.45 ^{i}	15.78 ± 0.52 ^{i}	14.67 ± 0.65 ^{i}	12.52 ± 0.29 ^{i}	17.20 ± 0.11 ^{i}	18.51 ± 0.10 ^{i}	24.47 ± 0.03 ^{i}	21.95 ± 0.23 ^{i}	22.72 ± 0.15 ^{i}
L. reuteri* + *cellulase	17.80 ± 0.49 ^{i}	15.47 ± 0.65 ^{i}	21.81 ± 0.21 ^{i}	20.18 ± 0.18 ^{i}	17.08 ± 0.13 ^{i}	23.79 ± 0.12 ^{i}	25.53 ± 0.43 ^{i}	33.77 ± 0.51 ^{i}	30.22 ± 0.44 ^{i}	31.60 ± 0.46 ^{i}
L. reuteri* + *Clara-diastase	20.85 ± 0.33 ^{i}	17.94 ± 0.57 ^{i}	25.35 ± 0.40 ^{i}	23.44 ± 0.18 ^{i}	19.76 ± 0.29 ^{i}	27.37 ± 0.48 ^{i}	29.47 ± 0.47 ^{i}	39.28 ± 0.18 ^{i}	35.11 ± 0.09 ^{i}	36.56 ± 0.12 ^{i}
L. reuteri* + *Viscozyme® L	19.19 ± 0.12 ^{i}	16.30 ± 0.30 ^{i}	23.61 ± 0.52 ^{i}	21.53 ± 0.65 ^{i}	18.34 ± 0.47 ^{i}	25.46 ± 0.20 ^{i}	27.24 ± 0.28 ^{i}	36.06 ± 0.22 ^{i}	32.20 ± 0.12 ^{i}	33.51 ± 0.08 ^{i}

B. animalis* + *cellulase	15.41 ± 0.49 i	13.18 ± 0.24 i	18.86 ± 0.20 i	17.10 ± 0.22 i	14.56 ± 0.31 i	20.31 ± 0.38 i	21.88 ± 0.13 i	29.16 ± 0.48 i	25.83 ± 0.45 i	26.86 ± 0.26 i
B. animalis* + *Clara-diastase	17.67 ± 0.25 i	14.92 ± 0.64 i	21.45 ± 0.20 i	19.50 ± 0.10 i	16.60 ± 0.12 i	22.90 ± 0.24 i	24.82 ± 0.27 i	33.02 ± 0.31 i	29.35 ± 0.12 i	30.66 ± 0.12 i
B. animalis* + *Viscozyme® L	16.29 ± 0.39 i	13.70 ± 0.44 i	19.69 ± 0.27 i	18.03 ± 0.45 i	15.28 ± 0.19 i	21.42 ± 0.35 i	22.89 ± 0.16 i	30.35 ± 0.07 i	27.25 ± 0.45 i	28.25 ± 0.11 i
L. acidophilus* + *cellulase	18.91 ± 0.41 i	15.70 ± 0.61 i	22.64 ± 0.65 i	20.88 ± 0.39 i	17.71 ± 0.23 i	24.55 ± 0.06 i	26.30 ± 0.25 i	34.94 ± 0.13 i	31.38 ± 0.37 i	32.55 ± 0.06 i
L. acidophilus* + *Clara-diastase	21.52 ± 0.22 i	18.27 ± 0.22 i	26.37 ± 0.39 i	24.18 ± 0.35 i	20.50 ± 0.41 i	28.32 ± 0.37 i	30.59 ± 0.45 i	40.48 ± 0.14 i	36.19 ± 0.24 i	37.67 ± 0.58 i
L. acidophilus* + *Viscozyme® L	20.33 ± 0.27 i	17.41 ± 0.34 i	24.69 ± 0.33 i	22.63 ± 0.40 i	19.32 ± 0.13 i	26.76 ± 0.17 i	28.74 ± 0.07 i	38.14 ± 0.27 i	34.02 ± 0.05 i	35.45 ± 0.39 i
**Spontaneous + *cellulase***	6.88 ± 0.61 i	5.74 ± 0.16 i	8.40 ± 0.41 i	7.85 ± 0.51 i	6.48 ± 0.11 i	9.11 ± 0.47 i	9.67 ± 0.65 i	12.80 ± 0.22 i	11.14 ± 0.59 i	12.04 ± 0.38 i
**Spontaneous + *Clara-diastase***	8.29 ± 0.37 i	7.17 ± 0.17 i	10.33 ± 0.42 i	9.49 ± 0.33 i	7.95 ± 0.27 i	11.16 ± 0.07 i	12.03 ± 0.22 i	15.84 ± 0.27 i	14.15 ± 0.26 i	14.99 ± 0.24 i
**Spontaneous + *Viscozyme® L***	7.63 ± 0.23 i	6.72 ± 0.33 i	9.51 ± 0.10 i	8.99 ± 0.65 i	7.37 ± 0.15 i	10.29 ± 0.65 i	10.96 ± 0.46 i	14.56 ± 0.08 i	13.03 ± 0.35 i	13.62 ± 0.46 i
***Clara-diastase* + Spontaneous**	22.69 ± 0.25 i	19.50 ± 0.65 i	27.56 ± 0.47 i	25.41 ± 0.57 i	21.70 ± 0.56 i	25.52 ± 0.40 i	27.47 ± 0.08 i	36.06 ± 0.63 i	32.71 ± 0.24 i	34.05 ± 0.41 i
Clara-diastase* + *L. acidophilus	31.59 ± 0.40 i	26.90 ± 0.34 i	37.97 ± 0.61 i	35.31 ± 0.23 i	30.05 ± 0.41 i	31.19 ± 0.60 i	33.49 ± 0.16 i	44.37 ± 0.10 i	39.66 ± 0.37 i	41.40 ± 0.59 i
Clara-diastase* + *B. animalis	25.46 ± 0.51 i	21.67 ± 0.04 i	31.15 ± 0.65 i	28.64 ± 0.27 i	24.13 ± 0.59 i	28.80 ± 0.19 i	30.93 ± 0.19 i	40.90 ± 0.65 i	36.65 ± 0.08 i	38.32 ± 0.65 i
Clara-diastase* + *L. reuteri	23.38 ± 0.45 i	19.89 ± 0.13 i	28.64 ± 0.15 i	26.26 ± 0.37 i	22.33 ± 0.56 i	28.89 ± 0.29 i	30.98 ± 0.37 i	40.88 ± 0.26 i	36.46 ± 0.33 i	37.92 ± 0.19 i
Clara-diastase* + *L. rhamnosus	23.09 ± 0.09 i	19.52 ± 0.21 i	28.43 ± 0.25 i	25.87 ± 0.05 i	21.93 ± 0.30 i	27.95 ± 0.04 i	30.04 ± 0.16 i	39.83 ± 0.44 i	35.62 ± 0.26 i	37.10 ± 0.64 i
***Viscozyme® L* + Spontaneous**	18.03 ± 0.20 i	15.21 ± 0.05 i	21.78 ± 0.25 i	19.93 ± 0.29 i	16.99 ± 0.15 i	23.68 ± 0.20 i	25.73 ± 0.42 i	33.69 ± 0.36 i	29.93 ± 0.61 i	31.29 ± 0.16 i
Viscozyme® L* + *L. acidophilus	26.06 ± 0.07 i	22.14 ± 0.08 i	31.84 ± 0.19 i	28.94 ± 0.60 i	24.68 ± 0.31 i	34.39 ± 0.28 i	37.11 ± 0.22 i	48.96 ± 0.07 i	43.58 ± 0.50 i	45.64 ± 0.60 i

Viscozyme® L + B. animalis	21.33 ± 0.06 [i]	18.10 ± 0.51 [i]	26.04 ± 0.23 [i]	23.84 ± 0.37 [i]	18.25 ± 0.04 [i]	23.98 ± 0.38 [i]	25.43 ± 0.12 [i]	33.95 ± 0.41 [i]	30.11 ± 0.23 [i]	31.55 ± 0.41 [i]
Viscozyme® L + L. reuteri	18.22 ± 0.37 [i]	15.62 ± 0.27 [i]	22.25 ± 0.02 [i]	20.30 ± 0.25 [i]	17.51 ± 0.61 [i]	24.10 ± 0.41 [i]	25.87 ± 0.10 [i]	34.41 ± 0.65 [i]	30.95 ± 0.65 [i]	31.66 ± 0.33 [i]
Viscozyme® L + L. rhamnosus	17.97 ± 0.64 [i]	15.28 ± 0.47 [i]	22.13 ± 0.43 [i]	20.16 ± 0.14 [i]	17.11 ± 0.06 [i]	23.63 ± 0.32 [i]	25.72 ± 0.36 [i]	33.69 ± 0.35 [i]	30.26 ± 0.32 [i]	31.58 ± 0.17 [i]
Cellulase + **Spontaneous**	16.16 ± 0.65 [i]	14.43 ± 0.43 [i]	20.25 ± 0.51 [i]	18.55 ± 0.17 [i]	15.64 ± 0.11 [i]	21.80 ± 0.09 [i]	23.44 ± 0.05 [i]	30.97 ± 0.45 [i]	27.81 ± 0.62 [i]	28.92 ± 0.16 [i]
Cellulase + L. acidophilus	24.95 ± 0.33 [i]	20.96 ± 0.65 [i]	30.29 ± 0.06 [i]	27.94 ± 0.62 [i]	23.66 ± 0.24 [i]	32.77 ± 0.11 [i]	35.49 ± 0.39 [i]	33.38 ± 0.09 [i]	29.89 ± 0.65 [i]	31.08 ± 0.19 [i]
Cellulase + B. animalis	17.25 ± 0.33 [i]	14.33 ± 0.29 [i]	20.69 ± 0.16 [i]	18.81 ± 0.65 [i]	16.16 ± 0.44 [i]	22.47 ± 0.38 [i]	24.12 ± 0.38 [i]	29.62 ± 0.16 [i]	26.36 ± 0.31 [i]	27.57 ± 0.06 [i]
Cellulase + L. reuteri	16.92 ± 0.26 [i]	14.30 ± 0.04 [i]	20.51 ± 0.03 [i]	18.82 ± 0.05 [i]	15.94 ± 0.60 [i]	21.96 ± 0.65 [i]	24.04 ± 0.52 [i]	31.41 ± 0.52 [i]	29.83 ± 0.25 [i]	31.11 ± 0.14 [i]
Cellulase + L. rhamnosus	16.65 ± 0.42 [i]	14.15 ± 0.17 [i]	20.33 ± 0.14 [i]	18.71 ± 0.12 [i]	16.00 ± 0.59 [i]	22.17 ± 0.51 [i]	23.70 ± 0.13 [i]	31.36 ± 0.22 [i]	28.15 ± 0.65 [i]	29.26 ± 0.12 [i]

Note. Values are expressed as rutin equivalents (mg RUE/g bee pollen) and represent the mean of 10 technical replicate measurements per sample. "i" denotes a statistically significant increase ($p < 0.05$).

Table S4. Changes in inhibition of heat-induced bovine serum albumin denaturation of bee pollen samples from different regions of Lithuania after various treatment methods.

Treatment	Inhibition of heat-induced bovine serum albumin denaturation of bee pollen samples, mg SAE/g									
	Klaipėda county	Telšiai county	Tauragė county	Marijampolė county	Šiauliai county	Utena county	Panevėžys county	Kaunas county	Alytus county	Vilnius county
**Natural**	11.12 ± 0.43	8.69 ± 0.18	12.04 ± 0.16	11.86 ± 0.16	9.53 ± 0.47	11.14 ± 0.08	13.13 ± 0.03	17.68 ± 0.28	17.45 ± 0.27	18.81 ± 0.65
**Spontaneous fermentation**	13.83 ± 0.18 ^{i}	10.96 ± 0.35 ^{i}	15.25 ± 0.40 ^{i}	15.06 ± 0.18 ^{i}	12.32 ± 0.14 ^{i}	14.42 ± 0.29 ^{i}	16.73 ± 0.49 ^{i}	19.91 ± 0.31 ^{i}	20.65 ± 0.15 ^{i}	22.08 ± 0.05 ^{i}
**Fermentation with *L. rhamnosus***	17.56 ± 0.37 ^{i}	13.67 ± 0.37 ^{i}	19.52 ± 0.49 ^{i}	18.87 ± 0.17 ^{i}	15.55 ± 0.41 ^{i}	18.37 ± 0.25 ^{i}	20.95 ± 0.28 ^{i}	29.22 ± 0.53 ^{i}	27.59 ± 0.27 ^{i}	29.53 ± 0.37 ^{i}
**Fermentation with *L. reuteri***	19.60 ± 0.45 ^{i}	15.64 ± 0.41 ^{i}	22.21 ± 0.29 ^{i}	21.35 ± 0.23 ^{i}	17.40 ± 0.30 ^{i}	20.80 ± 0.12 ^{i}	22.99 ± 0.22 ^{i}	33.17 ± 0.46 ^{i}	31.16 ± 0.64 ^{i}	33.12 ± 0.34 ^{i}
**Fermentation with *B. animalis***	17.92 ± 0.25 ^{i}	14.05 ± 0.26 ^{i}	20.23 ± 0.59 ^{i}	19.31 ± 0.13 ^{i}	15.70 ± 0.11 ^{i}	18.74 ± 0.09 ^{i}	21.36 ± 0.32 ^{i}	29.93 ± 0.22 ^{i}	28.30 ± 0.30 ^{i}	29.87 ± 0.29 ^{i}
**Fermentation with *L. acidophilus***	21.64 ± 0.39 ^{i}	17.14 ± 0.65 ^{i}	24.94 ± 0.05 ^{i}	23.78 ± 0.28 ^{i}	19.32 ± 0.43 ^{i}	23.15 ± 0.25 ^{i}	26.09 ± 0.29 ^{i}	36.95 ± 0.15 ^{i}	34.69 ± 0.33 ^{i}	37.27 ± 0.43 ^{i}
**Enzymatic hydrolysis with cellulase**	23.58 ± 0.34 ^{i}	18.57 ± 0.18 ^{i}	26.99 ± 0.65 ^{i}	25.55 ± 0.16 ^{i}	20.86 ± 0.10 ^{i}	25.03 ± 0.23 ^{i}	28.09 ± 0.65 ^{i}	40.04 ± 0.34 ^{i}	37.21 ± 0.52 ^{i}	39.74 ± 0.29 ^{i}
**Enzymatic hydrolysis with *Clara-diastase***	31.36 ± 0.54 ^{i}	25.05 ± 0.45 ^{i}	36.67 ± 0.44 ^{i}	34.30 ± 0.63 ^{i}	28.18 ± 0.15 ^{i}	34.12 ± 0.29 ^{i}	37.73 ± 0.65 ^{i}	54.69 ± 0.23 ^{i}	50.23 ± 0.65 ^{i}	53.59 ± 0.47 ^{i}
**Enzymatic hydrolysis with *Viscozyme® L***	25.95 ± 0.02 ^{i}	20.59 ± 0.16 ^{i}	29.89 ± 0.10 ^{i}	28.26 ± 0.58 ^{i}	23.10 ± 0.33 ^{i}	28.05 ± 0.37 ^{i}	30.99 ± 0.63 ^{i}	44.61 ± 0.30 ^{i}	41.33 ± 0.05 ^{i}	44.05 ± 0.24 ^{i}
L. rhamnosus* + *cellulase	26.91 ± 0.19 ^{i}	21.28 ± 0.06 ^{i}	31.05 ± 0.38 ^{i}	29.26 ± 0.33 ^{i}	23.56 ± 0.65 ^{i}	28.82 ± 0.03 ^{i}	32.19 ± 0.05 ^{i}	39.32 ± 0.51 ^{i}	36.49 ± 0.45 ^{i}	38.98 ± 0.10 ^{i}
L. rhamnosus* + *Clara-diastase	38.96 ± 0.33 ^{i}	30.92 ± 0.10 ^{i}	45.21 ± 0.65 ^{i}	42.57 ± 0.15 ^{i}	34.81 ± 0.51 ^{i}	42.33 ± 0.08 ^{i}	46.82 ± 0.46 ^{i}	57.87 ± 0.64 ^{i}	53.30 ± 0.26 ^{i}	56.64 ± 0.14 ^{i}
L. rhamnosus* + *Viscozyme® L	29.35 ± 0.31 ^{i}	23.36 ± 0.49 ^{i}	33.70 ± 0.49 ^{i}	31.76 ± 0.48 ^{i}	26.03 ± 0.60 ^{i}	31.73 ± 0.42 ^{i}	35.42 ± 0.33 ^{i}	43.10 ± 0.22 ^{i}	39.93 ± 0.06 ^{i}	42.61 ± 0.11 ^{i}
L. reuteri* + *cellulase	28.37 ± 0.28 ^{i}	22.60 ± 0.25 ^{i}	33.01 ± 0.46 ^{i}	31.07 ± 0.32 ^{i}	25.15 ± 0.46 ^{i}	30.32 ± 0.65 ^{i}	34.09 ± 0.65 ^{i}	48.87 ± 0.19 ^{i}	44.95 ± 0.50 ^{i}	48.06 ± 0.09 ^{i}
L. reuteri* + *Clara-diastase	43.79 ± 0.34 ^{i}	34.46 ± 0.43 ^{i}	50.89 ± 0.35 ^{i}	47.71 ± 0.50 ^{i}	39.11 ± 0.26 ^{i}	47.76 ± 0.28 ^{i}	52.58 ± 0.10 ^{i}	64.75 ± 0.41 ^{i}	59.03 ± 0.40 ^{i}	62.68 ± 0.05 ^{i}

L. reuteri* + *Viscozyme® L	33.59 ± 0.30 [i]	26.88 ± 0.65 [i]	39.00 ± 0.41 [i]	36.54 ± 0.35 [i]	29.95 ± 0.42 [i]	36.35 ± 0.39 [i]	40.39 ± 0.10 [i]	58.26 ± 0.33 [i]	53.51 ± 0.65 [i]	56.84 ± 0.17 [i]
B. animalis* + *cellulase	25.00 ± 0.07 [i]	19.94 ± 0.52 [i]	28.87 ± 0.48 [i]	27.22 ± 0.25 [i]	22.44 ± 0.24 [i]	26.91 ± 0.44 [i]	29.83 ± 0.60 [i]	42.95 ± 0.08 [i]	39.83 ± 0.41 [i]	42.52 ± 0.18 [i]
B. animalis* + *Clara-diastase	40.17 ± 0.61 [i]	31.77 ± 0.41 [i]	46.92 ± 0.65 [i]	44.10 ± 0.64 [i]	35.81 ± 0.20 [i]	43.59 ± 0.09 [i]	48.12 ± 0.20 [i]	55.73 ± 0.45 [i]	50.89 ± 0.65 [i]	54.25 ± 0.16 [i]
B. animalis* + *Viscozyme® L	30.12 ± 0.52 [i]	23.77 ± 0.12 [i]	34.76 ± 0.56 [i]	32.86 ± 0.15 [i]	26.77 ± 0.14 [i]	32.48 ± 0.30 [i]	35.98 ± 0.20 [i]	52.00 ± 0.65 [i]	47.86 ± 0.27 [i]	50.84 ± 0.21 [i]
L. acidophilus* + *cellulase	32.87 ± 0.65 [i]	26.24 ± 0.09 [i]	38.55 ± 0.41 [i]	36.22 ± 0.22 [i]	29.62 ± 0.13 [i]	35.94 ± 0.08 [i]	39.87 ± 0.14 [i]	51.34 ± 0.63 [i]	47.49 ± 0.09 [i]	50.70 ± 0.65 [i]
L. acidophilus* + *Clara-diastase	40.31 ± 0.21 [i]	31.96 ± 0.44 [i]	47.09 ± 0.42 [i]	44.10 ± 0.12 [i]	36.04 ± 0.28 [i]	43.79 ± 0.16 [i]	48.53 ± 0.30 [i]	61.84 ± 0.19 [i]	56.51 ± 0.12 [i]	60.29 ± 0.23 [i]
L. acidophilus* + *Viscozyme® L	37.88 ± 0.15 [i]	30.01 ± 0.14 [i]	44.09 ± 0.36 [i]	41.37 ± 0.26 [i]	33.81 ± 0.16 [i]	41.15 ± 0.09 [i]	45.61 ± 0.12 [i]	55.77 ± 0.13 [i]	51.10 ± 0.23 [i]	54.29 ± 0.36 [i]
**Spontaneous + *cellulase***	16.85 ± 0.04 [i]	13.26 ± 0.03 [i]	18.90 ± 0.29 [i]	18.08 ± 0.62 [i]	14.77 ± 0.27 [i]	17.56 ± 0.22 [i]	20.18 ± 0.53 [i]	25.48 ± 0.12 [i]	24.38 ± 0.36 [i]	25.93 ± 0.07 [i]
**Spontaneous + *Clara-diastase***	21.25 ± 0.49 [i]	16.81 ± 0.65 [i]	24.16 ± 0.13 [i]	23.09 ± 0.12 [i]	18.91 ± 0.47 [i]	22.56 ± 0.50 [i]	25.31 ± 0.61 [i]	36.09 ± 0.26 [i]	33.58 ± 0.22 [i]	35.85 ± 0.04 [i]
**Spontaneous + *Viscozyme® L***	19.56 ± 0.34 [i]	15.26 ± 0.10 [i]	22.06 ± 0.18 [i]	21.18 ± 0.47 [i]	17.24 ± 0.37 [i]	20.52 ± 0.25 [i]	23.22 ± 0.36 [i]	32.71 ± 0.62 [i]	30.66 ± 0.29 [i]	32.94 ± 0.28 [i]
***Clara-diastase* + Spontaneous**	43.59 ± 0.48 [i]	34.67 ± 0.12 [i]	51.40 ± 0.44 [i]	47.76 ± 0.05 [i]	39.02 ± 0.05 [i]	47.76 ± 0.50 [i]	52.67 ± 0.65 [i]	63.39 ± 0.14 [i]	58.00 ± 0.40 [i]	61.83 ± 0.33 [i]
Clara-diastase* + *L. acidophilus	53.92 ± 0.44 [i]	42.93 ± 0.33 [i]	63.53 ± 0.07 [i]	59.23 ± 0.44 [i]	48.38 ± 0.30 [i]	58.95 ± 0.35 [i]	65.30 ± 0.65 [i]	70.79 ± 0.26 [i]	64.50 ± 0.30 [i]	68.66 ± 0.14 [i]
Clara-diastase* + *B. animalis	50.04 ± 0.37 [i]	39.75 ± 0.22 [i]	58.99 ± 0.59 [i]	54.80 ± 0.17 [i]	44.82 ± 0.19 [i]	54.91 ± 0.25 [i]	60.09 ± 0.52 [i]	64.21 ± 0.12 [i]	58.81 ± 0.23 [i]	62.64 ± 0.58 [i]
Clara-diastase* + *L. reuteri	45.50 ± 0.53 [i]	35.80 ± 0.37 [i]	53.09 ± 0.37 [i]	49.52 ± 0.05 [i]	40.39 ± 0.20 [i]	49.47 ± 0.12 [i]	54.49 ± 0.05 [i]	63.01 ± 0.24 [i]	57.61 ± 0.03 [i]	61.22 ± 0.37 [i]
Clara-diastase* + *L. rhamnosus	43.28 ± 0.14 [i]	34.13 ± 0.55 [i]	50.73 ± 0.44 [i]	47.40 ± 0.05 [i]	38.86 ± 0.21 [i]	47.21 ± 0.23 [i]	52.04 ± 0.30 [i]	60.18 ± 0.26 [i]	55.22 ± 0.19 [i]	58.75 ± 0.13 [i]
***Viscozyme® L* + Spontaneous**	32.09 ± 0.39 [i]	27.57 ± 0.37 [i]	37.47 ± 0.12 [i]	35.26 ± 0.05 [i]	28.97 ± 0.37 [i]	35.02 ± 0.56 [i]	38.78 ± 0.13 [i]	51.09 ± 0.21 [i]	47.10 ± 0.06 [i]	50.16 ± 0.15 [i]

Viscozyme® L + L. acidophilus	44.07 ± 0.48 ^{i}	34.94 ± 0.24 ^{i}	51.58 ± 0.14 ^{i}	48.17 ± 0.41 ^{i}	39.31 ± 0.65 ^{i}	48.06 ± 0.32 ^{i}	53.12 ± 0.43 ^{i}	66.38 ± 0.21 ^{i}	60.72 ± 0.30 ^{i}	64.58 ± 0.40 ^{i}
Viscozyme® L + B. animalis	40.01 ± 0.10 ^{i}	31.59 ± 0.51 ^{i}	46.82 ± 0.03 ^{i}	43.80 ± 0.61 ^{i}	35.56 ± 0.20 ^{i}	43.62 ± 0.52 ^{i}	48.08 ± 0.59 ^{i}	55.34 ± 0.51 ^{i}	50.96 ± 0.10 ^{i}	54.26 ± 0.31 ^{i}
Viscozyme® L + L. reuteri	37.84 ± 0.23 ^{i}	30.05 ± 0.35 ^{i}	44.22 ± 0.31 ^{i}	41.31 ± 0.19 ^{i}	33.77 ± 0.13 ^{i}	40.98 ± 0.65 ^{i}	45.43 ± 0.61 ^{i}	60.18 ± 0.59 ^{i}	55.26 ± 0.16 ^{i}	58.66 ± 0.41 ^{i}
Viscozyme® L + L. rhamnosus	34.90 ± 0.45 ^{i}	27.79 ± 0.48 ^{i}	40.99 ± 0.25 ^{i}	38.42 ± 0.65 ^{i}	31.37 ± 0.65 ^{i}	38.26 ± 0.57 ^{i}	42.30 ± 0.55 ^{i}	52.64 ± 0.17 ^{i}	48.29 ± 0.47 ^{i}	51.69 ± 0.46 ^{i}
***Cellulase +* Spontaneous**	26.04 ± 0.09 ^{i}	24.48 ± 0.49 ^{i}	29.94 ± 0.22 ^{i}	28.46 ± 0.22 ^{i}	25.42 ± 0.82 ^{i}	27.90 ± 0.16 ^{i}	31.27 ± 0.10 ^{i}	44.75 ± 0.17 ^{i}	41.47 ± 0.64 ^{i}	44.11 ± 0.65 ^{i}
Cellulase + L. acidophilus	39.87 ± 0.53 ^{i}	31.64 ± 0.26 ^{i}	46.78 ± 0.37 ^{i}	43.57 ± 0.39 ^{i}	35.76 ± 0.23 ^{i}	43.41 ± 0.12 ^{i}	48.12 ± 0.31 ^{i}	55.29 ± 0.30 ^{i}	50.83 ± 0.07 ^{i}	54.04 ± 0.39 ^{i}
Cellulase + B. animalis	32.76 ± 0.65 ^{i}	26.30 ± 0.44 ^{i}	38.02 ± 0.33 ^{i}	35.69 ± 0.19 ^{i}	29.27 ± 0.46 ^{i}	35.35 ± 0.38 ^{i}	39.19 ± 0.65 ^{i}	46.92 ± 0.13 ^{i}	43.37 ± 0.30 ^{i}	46.24 ± 0.16 ^{i}
Cellulase + L. reuteri	33.97 ± 0.33 ^{i}	26.93 ± 0.05 ^{i}	39.59 ± 0.08 ^{i}	37.16 ± 0.11 ^{i}	30.37 ± 0.27 ^{i}	36.75 ± 0.43 ^{i}	40.91 ± 0.36 ^{i}	52.83 ± 0.12 ^{i}	48.57 ± 0.44 ^{i}	51.95 ± 0.53 ^{i}
Cellulase + L. rhamnosus	31.37 ± 0.58 ^{i}	24.83 ± 0.13 ^{i}	36.40 ± 0.65 ^{i}	34.09 ± 0.65 ^{i}	28.06 ± 0.34 ^{i}	33.95 ± 0.52 ^{i}	37.68 ± 0.51 ^{i}	46.48 ± 0.65 ^{i}	42.90 ± 0.11 ^{i}	45.54 ± 0.34 ^{i}

Note. Values are expressed as salicylic acid equivalents (mg SAE/g bee pollen). Values represent the mean of 10 technical replicate measurements per sample ("i" denotes a statistically significant increase, $p < 0.05$).
